# How Hydromorphological Pressures Co-govern Lake Water Quality in a Mediterranean Environment. A Study for Greek Natural Lakes and Reservoirs

**DOI:** 10.1007/s00267-026-02627-6

**Published:** 2026-09-17

**Authors:** Dionissis Latinopoulos, Chrysoula Ntislidou, Alexandra Carriere, Dimitra Kemitzoglou, Vasiliki Tsiaoussi, Ifigenia Kagalou

**Affiliations:** 1 Department of Civil Engineering, School of Engineering, Democritus University of Thrace, Xanthi, Greece; 2 Department of Zoology, School of Biology, Aristotle University of Thessaloniki, Thessaloniki, Greece; 3 ESAIP, CERADE, 18, rue du 8 mai 1945, 49180 St-Barthélemy d’Anjou, Cedex, France; 4 National Museum of Natural History Goulandris / Greek Biotope-Wetland Centre (EKBY), Thermi, Greece

**Keywords:** Water quality, Hydrological features, Morphological attributes, Landscape metrics, Biological quality elements (BQEs), Ecohydrological management.

## Abstract

Hydromorphological (HyMo) conditions are increasingly recognized as key drivers of ecological quality in lakes and reservoirs, yet their role in Mediterranean systems remains poorly understood. We hypothesize that the relations between HyMo (natural or anthropogenic) metrics and quality elements (biological and physicochemical; QEs) could be quantified to benefit lake functioning and ecological quality. This attempt to quantify HyMo and QEs links, is the first for Mediterranean lakes, and aims to identify the level of QEs responsiveness to specific HyMo metrics. National monitoring data from 24 natural lakes and 26 reservoirs across Greece (2012–2021) were used. A systematic screening procedure ended with seven and eleven metrics for natural lakes and reservoirs, respectively. Redundancy Analysis and stepwise multiple linear regression were applied to identify and quantify the influence of HyMo metrics on QEs. In natural lakes, mean depth and water level were the most consistent predictors, reflecting self-purification capacity and resilience to eutrophication, while non-natural land cover and agricultural land use negatively affected phytoplankton, macrophytes, and water transparency. In reservoirs, land cover characteristics (i.e., intensive agriculture and fallow land) within the 100 m buffer zone emerged as dominant drivers of phytoplankton and Chl-a variability. Principal Component Analysis revealed four distinct typological groupings for both system types, reflecting the interplay between morphological and land use metrics. These findings confirm that HyMo functioning is a significant mediator of ecological quality in Mediterranean lakes and reservoirs, supporting the integration of system-specific HyMo metrics into Water Framework Directive assessment and ecohydrological management strategies.

## Introduction

For decades, people have modified aquatic environments to facilitate various utilitarian objectives, including agriculture, navigation, energy production, settlements, and protection from floods (Naiman and Dudgeon 2011). Interventions for various water uses (e.g, water supply, flood protection) and land reclamation, have significantly transformed European natural lakes (Argillier et al. 2025). Consequently, these ecohydrological factors led to changes in morphological conditions (e.g., alteration of lake shorelines, littoral and riparian habitats), shifts in hydrological dynamics (e.g., dams’ construction, changes in water levels, reduced hydrological connectivity), and also altered water quality, ultimately impacting the ecological balance and biodiversity. These changes, collectively termed as hydromorphological (HyMo) alterations, impair aquatic ecosystem functioning, and restoration depends largely on returning HyMo conditions to more natural states (Kampa and Bussettini 2018). Good HyMo functioning stands as a fundamental pillar of ecosystem health and provides ecosystem services and societal benefits (Argillier et al. 2022). This is acknowledged by the Water Framework Directive (WFD, 2000/60/EC), which mandates an assessment of HyMo conditions for ecological status classification.

The characterization of lake hydromorphology plays a vital role for understanding the dynamics of the biodiversity they harbor (Kampa et al. 2019) and for that it is designated as a quality element (QE) supporting the assessment of the ecological status (or potential) of water bodies. HyMo pressures are determinant factors, especially in cases where good status cannot be achieved despite the measures taken (Poikane et al. 2020). When significant alterations in HyMo conditions are identified, monitoring and assessment systems and measures to address them are deemed necessary. In addition, hydromorphological (HyMo) restoration actions are already being implemented in several European countries, particularly in lake littoral zones, reflecting increasing recognition of their role in improving ecological conditions (Garron et al. 2024). To assess HyMo conditions, it is also necessary to examine the correlation between biological QEs (BQEs) and nutrient components according to complex methodologies. All BQEs are influenced based on their ecological requirements by hydromorphology; and in turn reflect specific pressures (mainly eutrophication) in certain microhabitats (Kutyła et al. 2025). In addition to the parameters recommended by the WFD for assessing the hydrological and morphological status of lakes, Argillier et al. (2022) suggest a framework to encompass six key hydrological and seven morphological aspects, which describe the quantity and dynamics of the flow and littoral, shore, bed and bank attributes, that govern lake profile/functioning, all in compliance with the ECOSTAT report (Birk and Kampa 2018).

The wide picture of the European Union (EU) demonstrates that 54% of surface waters across Europe are subject to notable HyMo pressures (WISE 2024a). In Greece, until 2012, parameters such as water abstraction and flow regulation were not classified as HyMo pressures. According to the third revision of River Basin Management Plans (RBMPs), 30 lakes (45% of all lakes, 47% of total lake area) exhibit HyMo alterations, making them the second source of pressures, following diffuse pollution that cover 70% of total lake area (WISE 2024a). Furthermore, 58% of lake water bodies in Greece appear not to achieve good ecological status (WISE 2024b).

Many WFD monitoring and assessment methods are broad and incorporate multiple metrics, which often respond weakly to specific HyMo pressures. This can be improved by creating more targeted indicators. For instance, morphological changes could be assessed with methods like Lake Habitat Survey (LHS, Rowan et al. 2006) either alone (Latinopoulos et al. 2018) or combined with biological data (Miller et al. 2013). Several Member States already utilize such targeted indicators within their assessment systems (e.g., Carriere et al. 2024). HyMo pressures influence BQEs through the morphological processes that affect habitat quality and structure, with riparian vegetation typically playing a significant role. A better characterization and ecohydrological quantitative description of the connection between HyMo alterations and their impact on biological parameters are necessary, utilizing appropriate data and targeted indicators (Poikane 2020).

The relationship between hydromorphology and biological elements is complex and a simple linear correspondence cannot be expected. It requires a comprehensive understanding of the parameters and processes involved in each case. One issue observed is that favorable HyMo conditions often do not impact BQEs reliant on pollutants and other physicochemical factors (Carriere et al. 2024). Organisms’ susceptibility to external perturbation, ecology and reaction time may govern their response by altering taxa representation in communities’ composition (Argillier et al. 2025). Consequently, significant ecohydrological gaps exist in understanding how HyMo pressures influence biological communities, because their impacts on HyMo processes and habitat conditions are not consistently integrated into assessment methods (Bussettini et al. 2018). In most countries, the connection between HyMo alterations and BQEs has not been evaluated (Birk and Kampa 2018). Therefore, there is a need for the development of robust methodological approaches to investigate this relationship.

Our basic hypothesis is that HyMo metrics, natural or anthropogenic, affect QEs in different ways and their relations may act as a catalyst for either “protecting” the lakes/reservoirs by preserving lake functioning or multiplying the synergistic effect of pressures/stressors on ecological quality. The present study aims to quantify the relationships between HyMo metrics and both BQEs and supporting physicochemical QEs in Greek natural lakes and reservoirs, identifying which quality elements are most responsive to specific HyMo metrics. Moreover, we attempt to identify deficiencies in current HyMo assessment frameworks applicable to Greek water bodies, thereby contributing to Water Framework Directive (WFD) compliance and EU harmonization efforts. Thus, our first objective was to quantify both BQEs and supporting physicochemical QEs correlations with HyMo metrics. A secondary objective was to address HyMo gaps in Greek natural lakes and reservoirs and ultimately support the WFD requirements for EU harmonization, acknowledging the horizontal lag in EU compliance.

## Materials and Methods

### Study Area

**Fig. 1:**
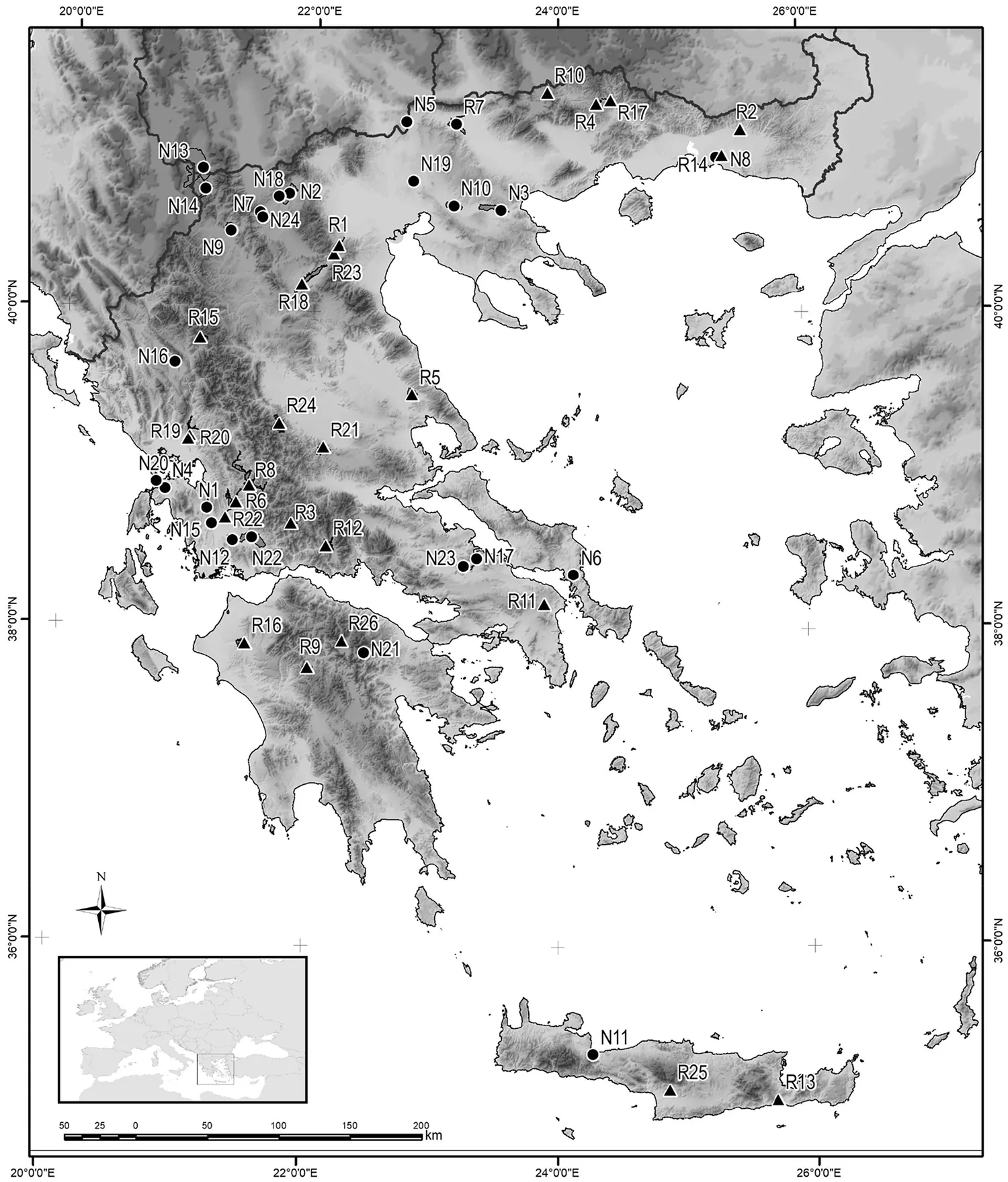
Lakes and reservoirs of Greece included in the assessment. Natural lakes are denoted by N and reservoirs by R. N1: Amvrakia, N2: Vegoritida, N3: Volvi, N4: Voulkaria N5: Doirani, N6: Distos, N7: Zazari, N8: Ismarida, N9: Kastoria, N10: Koronia, N11: Kourna, N12: Lysimachia, N13: Megali Prespa, N14: Mikri Prespa, N15: Ozeros, N16: Pamvotida, N17: Paralimni, N18: Petron, N19: Pikrolimni, N20: Saltini, N21: Stimfalia, N22: Trichonida, N23: Yliki, N24: Cheimaditida, R1: Asomaton, R2: Gratini, R3: Evinos, R4: Thisauros, R5: Karla, R6: Kastraki, R7: Kerkini, R8: Kremaston, R9: Ladona, R10: Leukogeion, R11: Marathonas, R12: Mornos, R13: Bramianon, R14: Adriani, R15: Pigon Aoou, R16: Pineios, R17: Platanovrisi, R18: Polifitou, R19: Pournariou, R20: Pournariou II, R21: Smokovo, R22: Stratos, R23: Sfikia, R24: Tavropos, R25: Faneromeni, R26: Feneou

The study area covers the entire Greek territory, a typical Mediterranean environment, with intense relief and relatively small waterbodies (Fig. 1). The Greek National Water Monitoring Network (JMD 140384/2011) comprises 50 lake water bodies, including both natural lakes and reservoirs. The key hydrological and morphological characteristics of the water bodies included in the present analysis are summarised in Table 1 and S1. The climatic profile is predominantly Mediterranean (from arid to semiarid) with temperate character for lakes in higher altitudes. There is an extended warm period (>15 ^o^C) from March until October (Lagouvardos et al. 2024). Most natural shallow lakes are polymictic while deeper ones are warm monomictic (Kagalou et al. 2021). Climate change has already affected hydrology (inflow patterns shaped by heavy rainfalls) and has also introduced prolonged droughts (Lagouvardos et al. 2024).

### Literature Review

We first examined and compared assessment criteria for HyMo alterations in lakes, drawing on: (a) the national methodology, (b) approaches used by other EU member states for WFD implementation, (c) recommendations from WFD Common Implementation Strategy working groups including Guidance Documents No. 10, 23 and 37, (d) guidance from ETC/ICM and the European Environment Agency, and (e) scientific publications proposing WFD-based methods. Specifically, from the europa.eu portal umbrella was searched: EEIONET (EEA Network 2025), EEA (EEA 2025), CIRCABC (CIRCABC 2025), Publications Office of the EU (Publications Office 2025) and CORDIS (CORDIS 2025). Selection criteria for methodological approaches included: applicability in a Mediterranean environment, number of parameters included in each method and their respective weightings, and ease of application in Greek lakes.

Relevant literature was identified through Scopus and Google Scholar searches using 16 keywords related to lake status, HyMo processes and pressures, hydrological and morphological alterations, groundwater connections, residence time, stratification, water-level regimes, aquatic vegetation, shoreline structure and substrates. References containing these terms were screened (scoping review) for inclusion. Specifically, a total of 78 references were identified from international literature from 2010 to 2025.

### Data Sources and HyMo Metric Development

The datasets was provided by the Greek Biotope/Wetland Center (EKBY; www.wfd.ekby.gr) covered a time range from 2012 to 2021 and included: (a) the values of ecological quality ratio (EQR) for phytoplankton (Tsiaoussi et al. 2016, 2017), macrophytes (Zervas et al. 2018) and benthic macroinvertebrates indices (Mavromati et al. 2021); (b) water transparency (Secchi Depth, SD - meters), pH, and dissolved oxygen (DO, mg L^–1^); (c) concentrations of total phosphorus (TP, μg L^-1^), chlorophyll-a (Chl-a, μg L^–1^), and nitrates (NO₃, mg L^-1^); (d) hydrological characteristics of Greek lakes, and (e) morphological parameters for natural lakes and reservoirs (Table 1). EQR values for phytoplankton, macrophytes, and benthic macroinvertebrates were imported as pre-calculated outputs from the national monitoring dataset provided by EKBY and were not derived or recalculated by the authors for the present study. Therefore, the analyses were conducted using a lake-year approach, where each observation corresponded to a specific lake each year. For each lake-year, EQR values and physicochemical variables were calculated as the mean of all available samples and monitoring stations within that year, thereby minimizing within-year temporal and spatial variability, following a similar aggregation approach applied in recent studies on Greek lakes (e.g., Nikolopoulou et al. 2025). The dataset was enriched with relevant literature, including shoreline habitat types (Mavromati et al. 2023), NATURA 2000 habitat classifications (EEA 2020), and Corine Land Cover data in different buffer zones (CORINE Land Cover 2018; Supplementary material, Table S2).

**Table 1:** Summary of hydromorphological characteristics of natural lakes and reservoirs included in the study (mean values calculated across sites; range in parentheses) based on the data available in Table S1

Variable Group	Environmental Variable (unit)	Natural lakes (*n* = 24)		Reservoirs (*n* = 26)	
		Mean	SD (range)	Mean	SD (range)
**Distribution**	Latitude (°N)	39.57	1.43 (35.33–41.22)	39.23	1.69 (35.04–41.40)
	Longitude (°E)	22.20	1.27 (20.77–25.32)	22.70	1.44 (20.99–25.70)
**Morphology**	Area (km²)	36.47	62.69 (0.56–281.68)	15.92	20.41 (0.46–66.67)
	Volume (x10^6^ m³)	356.21	681.18 (1.44 –2,739.13)	358.28	735.38 (0.95–3,453.78)
	Lake perimeter (m)	25,058	16,349 (3,360–57,595)	44,223	47,161 (3,662–211,273)
	Mean depth (m)	9.49	9.34 (0.81–29.33)	19.71	12.14 (0.95–46.27)
**Hydrology**	Retention time (years)	6.32	8.32 (1.00–35.10)	1.18	1.00 (0.01–4.58)
	Absolute elevation of water level (m a.s.l)	253.90	306.22 (0.32–849.60)	308.95	315.52 (4.28–1,339)
**Catchment**	Catchment area (km²)	453.00	613.33 (10.80–2,403)	1,278	2,542 (6.50–11,599)
	Schindler’s ratio (-, catchment area to volume)	29.28	47.81 (3.67–203.61)	75.57	73.96 (7.85–326.71)

From the published metrics identified in the literature review, the existing Greek-lake dataset covered 15 hydrological and morphological metrics for most of both natural lakes and reservoirs. This number was considered restrictive, prompting the combination of available information to develop meaningful and ecologically relevant metrics. The aforementioned process led to the quantification of 24 morphological, 4 hydrological metrics, and 6 typological descriptors (Supplementary material, Table S2). Distinction of those metrics was applied depending on if those were affected by anthropogenic activities or they derive naturally (Supplementary material, Table S2). In some cases, a clear distinction was not possible due to the nature of the water body (natural lakes/reservoir) and/or the magnitude of the modifications (e.g., 14.4 natural land cover in shoreline).

### Statistical Analyses

#### Selection of HyMo Metrics

A total of 115 HyMo metrics were initially considered for natural lakes (103 morphological and 12 hydrological) and 46 for reservoirs (35 morphological and 11 hydrological), and subsequently screened to identify the most appropriate ones. To reduce multicollinearity among HyMo metrics, Spearman rank correlation was applied using a threshold of rs >0.8. As a result, 46 morphological and 4 hydrological metrics were removed for natural lakes (Supplementary material, Figs. S1 and S2) and 13 morphological and 10 hydrological metrics for reservoirs (Supplementary material, Figs. S3 and S4). Correlations were visualized using the “corrplot” package in R (Wei and Simko 2017). Box plots were then used to identify and exclude narrow value ranges, irregular distributions, zero values, or extreme outliers (Hering et al. 2006), resulting in the removal of an additional 52 morphological and 6 hydrological metrics for natural lakes (Supplementary material, Fig. S5) and 12 morphological metrics for reservoirs (Supplementary material, Fig. S6). The combined screening procedure resulted in the selection of 7 and 11 HyMo metrics for natural lakes and reservoirs, respectively (Table 2). Then, continuous variables were log(x + 1)-transformed to reduce right skewness and heteroscedasticity, while percentage variables were arcsine square root-transformed to stabilise variance of bounded proportional data. All analyses were conducted using the statistical software package SPSS v28.

**Table 2:** Hydromorphological metrics retained for natural lakes (N) and reservoirs (R) after screening for multicollinearity and distributional adequacy. Land use percentages are derived from the Corine Land Cover (CLC 2018) dataset within a 100 m buffer zone

Metrics	Description	N	R
Dmax_macro	Maximum colonization depth of macrophytes	✓	
Per_Unmod_shore	Percentage of shoreline complexity and modification, anthropogenic use of littoral areas – Unmodified	✓	
Per_agri	CLC—Arable land (%)	✓	
Per_non_natural	Non-natural land cover (%) within the 100 m buffer zone	✓	
Per_macro_veg	Coverage of macrophytes in the littoral zone	✓	
mDepth	Mean depth	✓	✓
Wlevel	Absolute elevation of water level	✓	✓
Retention	Retention time		✓
mDepth_area	Mean depth/Lake area		✓
Nb_natural_100	Number of recorded natural land cover types (100 m)		✓
Per_Int_Agri_100	Percentage of intensive agriculture area (100 m)		✓
Per_Area_basin	Lake area/catchment area		✓
Per_Fallow	CLC—Fallow land (%)		✓
Per_Fore	CLC—Forests (%)		✓
Per_shrubs	CLC—Shrub and/or herbaceous vegetation associations (%)		✓
Per_low_veg	CLC—Open spaces with little or no vegetation (%)		✓

✓ indicates inclusion in the respective water body

#### Environmental Gradient Analysis

The relationships between HyMo metrics and biological and physicochemical QEs were examined using gradient analysis in CANOCO v4.5.1 (ter Braak et al. 2002). Detrended Correspondence Analysis (DCA) was first applied to determine whether biological and physicochemical data showed linear or non-linear responses to HyMo metrics. If the gradient length of the first axis was less than three times the standard deviation of the biological and physicochemical variables, a linear response was assumed, and Redundancy Analysis (RDA) was applied. Otherwise, if the gradient length exceeded this threshold, a non-linear response was assumed, and Canonical Correspondence Analysis (CCA) was used. The response matrix comprised EQR values of biological quality elements (phytoplankton, macrophytes, benthic macroinvertebrates) and physicochemical parameters (TP, Chl-a, Secchi depth, DO, pH, NO_3_), with rows representing individual water bodies and columns representing QE variables. The explanatory matrix contained the HyMo metrics for the same water bodies. Significant HyMo metrics influencing biological and physicochemical variables were identified using a Monte Carlo permutation test (*p* < 0.05) with 999 unrestricted permutations under the full model. Both the first constrained axis and all constrained axes combined were tested for significance. Additionally, the variation inflation factor (VIF) was assessed to detect multicollinearity; and metrics with VIF > 20 (Ter Braak 1986) were removed from the analysis. Lakes and reservoirs with incomplete data across all metrics were excluded from the above analysis to ensure data integrity. Specifically, natural lakes Doirani, Distos, Koronia, Pikrolimni, Saltini, and Stimfalia, as well as the reservoirs Gratini, Ladona, Adriani, Smokovo, Karla, Kerkini, Leukogeion, Pournariou II, and Stratos were excluded from further analysis.

To further quantify the relative contribution of different groups of hydromorphological predictors to the variance in biological and physicochemical QEs, variation partitioning analysis was performed separately for natural lakes and reservoirs using the ‘vegan’ package (Oksanen et al. 2022) in R (R Core Team 2023). Following the classification described in Table S2, the HyMo metrics retained after the screening procedure were assigned to three predictor groups according to morphological, hydrological, and land uses metrics. Adjusted R² values were used to quantify the unique and shared contributions of each predictor group to the total explained variance, with negative adjusted R² values set to zero following standard practice (Borcard et al. 1992). The statistical significance of each unique fraction was assessed using permutation tests with 999 unrestricted permutations.

#### Regression Modeling of HyMo Effects

To assess the influence of HyMo metrics on biological and physicochemical QEs, a stepwise linear regression analysis was conducted for the selected metrics that derived from 2.4.1 section. The general form of the multiple linear regression model applied was:

1 $$Y \sim \alpha +1{\beta }_{1}{X}_{1}+{\beta }_{2}{X}_{2}+{\beta }_{3}{X}_{3}+\ldots +{\beta }_{v}{X}_{v}$$

where *α*: the intercept, *β*_1_, *β*_2_, *β*_3_, …, *β*_n_: the slope coefficients, *X*: the explanatory variables (HyMo metrics), and *Y*: the dependent variable (biological or physicochemical QEs).

Stepwise regression (forward, backward, and bidirectional) was used to develop the Minimum Adequate Model (MAM), optimized using the lowest Akaike (AIC) and Bayesian (BIC) Information Criteria. Model assumptions were tested using the “gvlma” package in R (RStudio 2022, v4.2.2), assessing: (i) y linearity (Global Test), (ii) normality (Skewness-Kurtosis), (iii) heteroscedasticity, (iv) whether the explanatory variables are continuous or categorical, at a significance level of p = 0.05. Multicollinearity was tested VIF ( < 10) using the “DAAG” package (Tu et al. 2005). Finally, multiple linear regressions with adjusted R^2^ values < 0.4 and *p* > 0.05, as well as those in which the dependent variable was not significantly associated (*p* > 0.05) with a predictor, were excluded from further consideration. To assess the relative importance of each predictor in the regression models, the contribution of each variable was quantified using the proportion of its absolute t-value relative to the sum of absolute t-values of all predictors in the model (|t| / Σ|t| × 100) (Johnson and LeBreton 2004).

#### Identification of Key HyMo Features Impacting Lake Functioning

Prior to PCA, all variables were transformed as described above. No additional standardization was applied as transformations ensured comparability across variables. Principal Component Analysis (PCA) was applied to identify the primary HyMo metrics contributing most significantly to the overall variance in both natural lakes and reservoirs in a Mediterranean region. The software Primer V6 (Clarke and Gorley 2006) was utilized to conduct the analysis. Due to data gaps, natural lakes Doirani, Distos, Koronia, Pikrolimni, Saltini, and Stimfalia, as well as the reservoirs Gratini, Ladona, Adriani, Smokovo, Karla, Kerkini, Leukogeion, Pournariou II and Stratos were not considered.

## Results

### Environmental Gradient Analysis

**Fig. 2:**
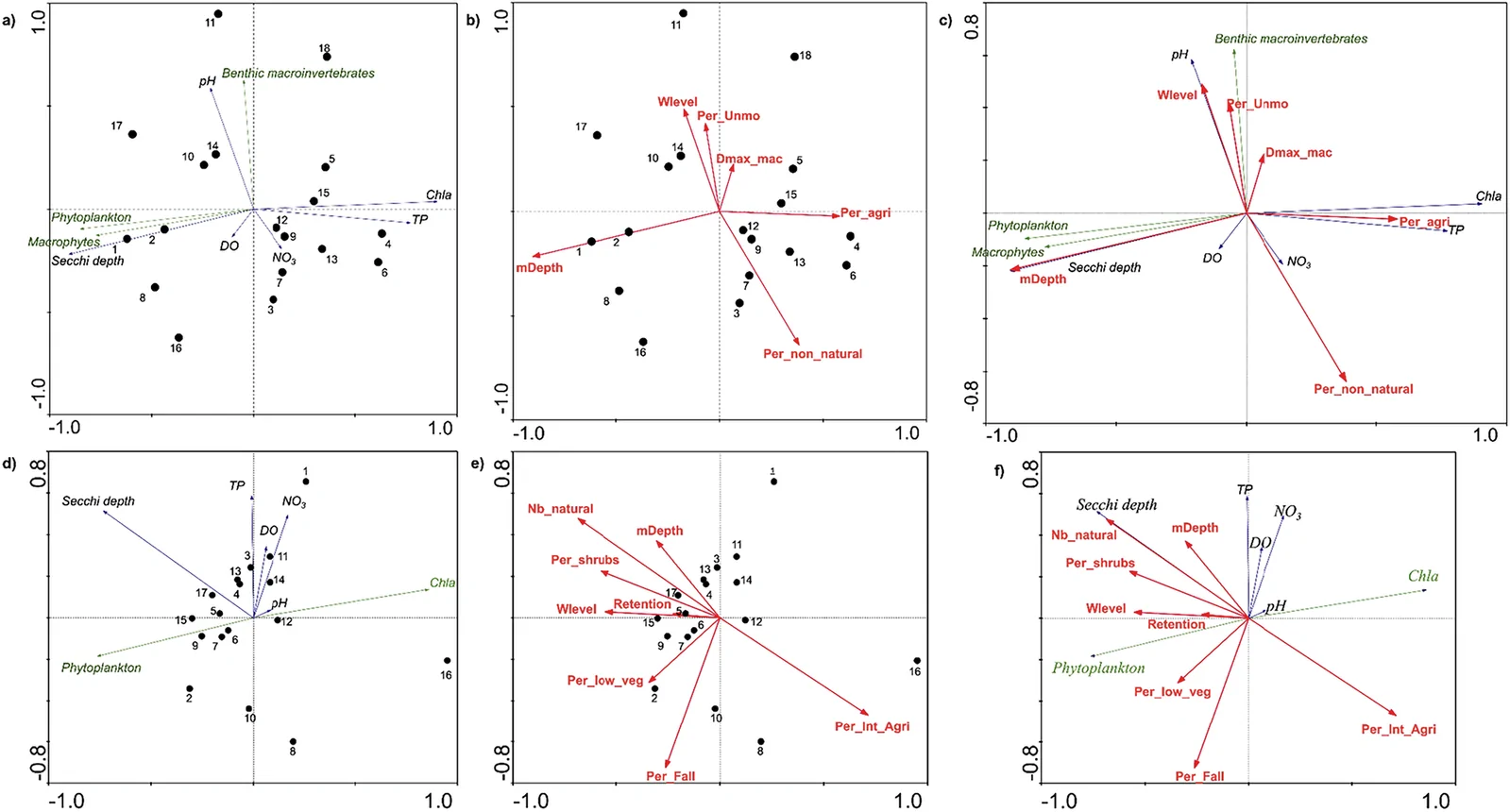
Redundancy Analysis for natural lakes and reservoirs: **a** and **d** biological and physicochemical quality elements (QEs), **b** and **e** hydromorphological (HyMo) metrics and **c** and **f** biological-physicochemical QEs and HyMo metrics. Black dots and numbers represent natural lakes and reservoirs (numbering as in Figs. 4 and 5, respectively). BQEs are shown as green arrows, physicochemical parameters as blue arrows, and HyMo metrics as red arrows. Maximum colonization depth of aquatic macrophytes (Dmax_macro), percentage of complexity and modification of the shoreline, anthropogenic use of riparian areas—unmodified (Per_Unmod_shore), percentage of agricultural land (Per_agri), percentage of non-natural land cover (Per_non_natural), mean depth (mDepth), absolute elevation level (Wlevel, number of natural land cover types (Nb_natural_100), percentage of intensive agriculture (Per_Int_Agri_100), percentage of land cover by fallow areas (Per_Fallow), retention time (Retention)

The DCA analysis confirmed linear responses of biological and physicochemical QEs for natural lakes (maximum gradient length: 0.616 SD units) and reservoirs (maximum gradient length: [value] SD units), justifying the use of RDA. Thus, **t**he RDA analysis (Table 3; Fig. 2) indicates that the first two axes explained 96.5% for natural lakes and 95.3% for reservoirs of the constrained variance between biological-physicochemical QEs and HyMo metrics (Table 3). For natural lakes, the Monte Carlo permutation test confirmed mean depth as the most statistically significant HyMo metric (*p* < 0.05), which appeared to be the most important factor influencing water quality changes and was positively associated with macrophytes and phytoplankton EQR values (Fig. 2a–c). Benthic invertebrates EQR values were negatively correlated with the percentage of non-natural land cover, while a positive association with NO_3_ concentrations was also observed (Fig. 2a–c). Phytoplankton and macrophytes EQR values were positively correlated with water transparency and negatively with TP and Chl-a (Fig. 2a). Especially, the latter (TP and Chl-a) were positively correlated with the percentage of arable land (Fig. 2c). Deep, high-transparency natural lakes, i.e., Amvrakia, Kourna and Trichonida, were positioned on the negative side of the Axis I, whereas shallower and eutrophication-impacted lakes, i.e., Voulkaria, Zazari and Ismarida, were associated with higher Chl-a and TP concentrations (Fig. 2a), characterized by the highest proportion of arable land (>69%; Fig. 2b).

**Table 3:** Cumulative percentage variance refers to the proportion of constrained (fitted) variance explained by each axis, i.e., the variance of the response matrix attributable to the HyMo metrics, rather than the total variance in the dataset

Water body	Axis	Cumulative percentage variance		Test of significance		Coefficients
		QEs (%)	QEs & HyMo metrics (%)	First axis	All axes	
**Natural lakes**	I	47.9	76.4	0.002	0.002	Dmax_mac (0.068); Per_Unmod_shore (–0.068); Per_agri (0.581); Per_non_natural (0.385); mDepth (–0.906); Wlevel (–0.172)
	II	60.6	96.5			Dmax_mac (0.226); Per_Unmod_shore (0.419); Per_agri (−0.023); Per_non_natural (−0.638); mDepth (−0.216); Wlevel (0.489)
**Reservoirs**	I	64.0	83.4	0.044	0.026	*mDepth (−0.305), *Retention (−0.226); Wlevel (−0.552); Nb_natural_100 ( −0.686); Per_Int_Agri (0.713); *Per_Fallow (−0.261); Per_shrubs (-0.575); *Per_low_veg (−0.342)
	II	73.2	95.3			mDepth (0.371), Retention (0.019); Wlevel (0.029); Nb_natural_100 (0.477); Per_Int_Agri (−0.469)*; Per_Fallow (−0.725)*; Per_shrubs (0.225); Per_low_veg (−0.311)

Coefficients represent the canonical coefficients of each hydromorphological variable on the respective RDA axis, indicating the direction and strength of its association with the biological and physicochemical qualitative elements. *Significant at p < 0.05

For reservoirs, the Monte Carlo permutation test identified Per_Int_Agri as the dominant gradient-structuring factor (0.713 on Axis I), Per_Fallow as the primary driver of Axis II (–0.725), and Wlevel as a secondary but statistically significant explanatory variable (–0.552 on Axis I). Natural land cover metrics (Nb_natural_100, Per_shrubs) were retained in the forward selection but represented accompanying patterns of lower individual explanatory power, acting as counterbalancing variables to the agricultural pressure gradient (Fig. 2d–f). A negative association was observed between the intensive agriculture and phytoplankton EQR values (Fig. 2d–f), while the fallow land cover displayed a negative correlation with the concentrations of NO_3_, TP, and DO along Axis II (Fig. 2d–f). This is exemplified by the positioning of the nitrogen polluted Asomaton reservoir and the intensively cultivated buffer zone of Faneromeni reservoir and the fallow practices of Bramianon and Pineios (Fig. 2e). Although water level was a statistically significant explanatory variable, its relative contribution to the constrained variance was comparatively lower than the dominant agricultural metrics, with canonical coefficients below 0.55 on a single axis.

**Fig. 3:**
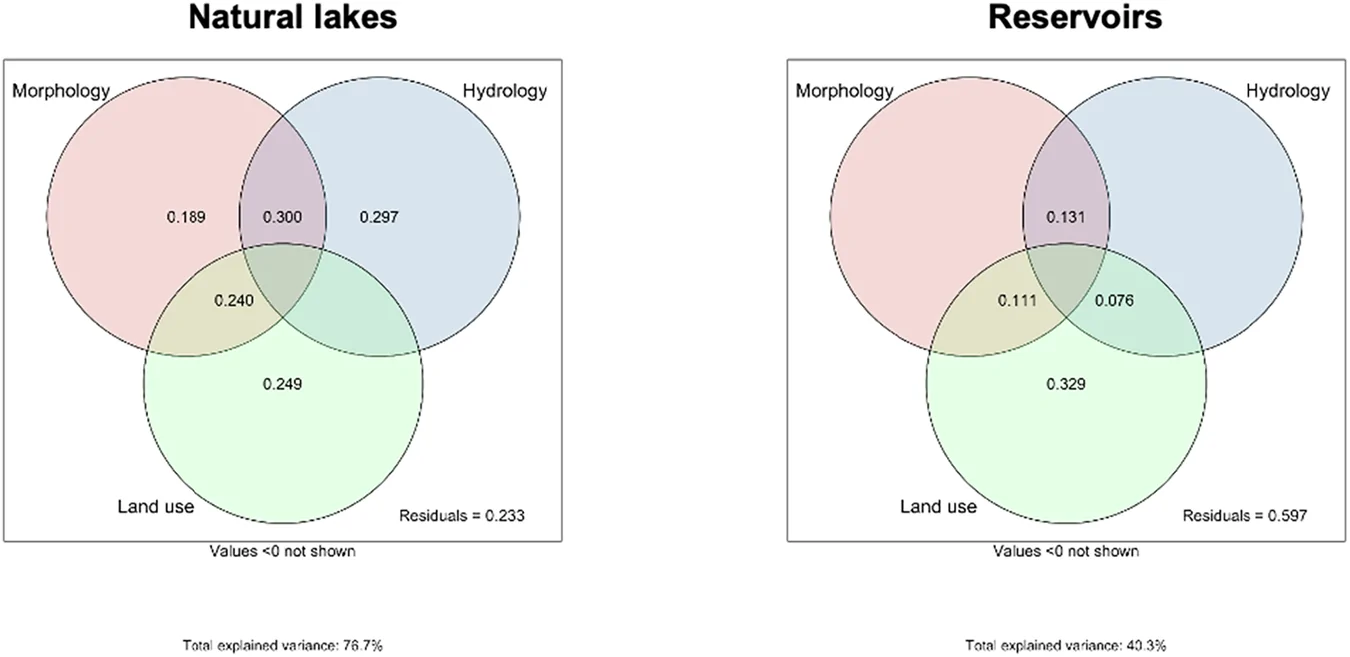
Variation partitioning of biological and physicochemical quality element (QE) variance among three predictor groups morphological (red), hydrological (blue), and land use (green) metrics for natural lakes and reservoirs. Residuals represent the proportion of variance not explained by any of the three predictor groups

Variation partitioning analysis revealed that for natural lakes the three groups jointly explained 76.7% of QE variance (adjusted *R*² =  0.767; Fig. 3). Hydrological metrics (water level) represented the only statistically significant unique fraction (adjusted *R*² = 0.297, *p* = 0.022), while morphological metrics (adjusted *R*² = 0.189, *p* = 0.057) and land use variables (adjusted R² = 0.249, *p* = 0.067) approached but did not reach significance. A substantial shared fraction between morphological and hydrological metrics (adjusted *R*² = 0.300) suggested collinearity between these groups, consistent with deeper lakes exhibiting distinct hydrological regimes. For reservoirs, the three groups jointly explained 40.3% of QE variance (adjusted *R*² = 0.403; Fig. 3). Land use pressure variables were the dominant predictor group (adjusted *R*² = 0.460, unique fraction = 0.329), while morphological and hydrological variables showed negative unique fractions, indicating their explanatory power largely overlapped with land use.

### Influence of Key HyMo Metrics on QEs

In natural lakes, each biological and physicochemical QE was modeled using multiple linear regression with seven HyMo metrics, as follows:

2 $${Quality}\,{element} \sim D\max {{{\rm{\_}}}}{macro}+{Per}{{{\rm{\_}}}}{Unmod}{{{\rm{\_}}}}{shore}\\ +{Per}{{{\rm{\_}}}}{agri}+{Per}{{{\rm{\_}}}}{non}{{{\rm{\_}}}}{natural}\\ +{Per}{{{\rm{\_}}}}{macro}{{{\rm{\_}}}}{veg} +{mDepth}+{Wlevel}$$

For macrophytes, metrics directly related to macrophyte characteristics, namely the maximum colonization depth of aquatic macrophytes (Dmax_macro) and the percentage coverage of aquatic vegetation (Per_macro_veg), were removed to avoid confounding effects. Concerning reservoirs, 11 metrics involved in the MAMs were specified by the following equation:

3 $${Quality}\,{element} \sim {mDepth}+{Retention}+{Wlevel}\\ +{mDepth}{{{\rm{\_}}}}{area}+{Nb}{{{\rm{\_}}}}{natural}{{{\rm{\_}}}}100\\ +{Per}{{{\rm{\_}}}}{Int}{{{\rm{\_}}}}{Agri}{{{\rm{\_}}}}100+{Per}{{{\rm{\_}}}}{Area}{{{\rm{\_}}}}{basin}\\ +{Per}{{{\rm{\_}}}}{Fallow}+{Per}{{{\rm{\_}}}}{Fore}+{Per}{{{\rm{\_}}}}{shrubs}\\ +{Per}{{{\rm{\_}}}}{low}{{{\rm{\_}}}}{veg}$$

Multiple linear regression in natural lakes identified lake morphology and hydrology as the most consistent predictors of water quality, with land use metrics providing additional explanatory power across several models (Table 4). Nitrate concentrations were not significantly predicted by any of the tested variables (adjusted *R*² = 0.140, *p* = 0.172; Table 4). The relative contribution of most models reflected multiple drivers, with Wlevel being the most influential in the phytoplankton (38.3%), DO (35.7%), and pH (25.1%) models and mDepth dominating the Chl-a (30.9%), Secchi depth (44.3%) and TP (100%) models. The TP model was the only single-predictor model in the dataset.

**Table 4:** Final models derived from stepwise [forward/backward/bidirectional] multiple linear regression analysis of biological and physicochemical quality elements with hydromorphological metrics for natural lakes and reservoirs

Metrics	Description
	Natural lakes
**Phyto**	**Phyto** = 2.266*** − **0.524 x** ***Dmax_macro*** + **0.349 x** Per_Unmodified_shore** − **0.345 x** Per_agri* − **0.539 x** Per_non_natural*** − **0.319 x** Wlevel*** **Multiple-R**^**2**^**: 0.924, Adjusted-R**^**2**^ = **0.877, p** = **0.0002; Relative contribution: Wlevel (38.3%), Per_non_natural (23.5%), Per_Unmodified_shore (15.8%), Per_agri (13.0%), Dmax_macro (9.4%)**
**Macro**	**Macro** = 1.920*** − **0.527 x** Per_non_natural* − **0.233 x** Wlevel* **Multiple R**^**2**^**: 0.537, Adjusted-R**^**2**^ = **0.453, p** = **0.014; Relative contribution: Wlevel (50%), Per_non_natural (50%)**
Benthos	Benthos= 1.058*** + 0.257 x *Per_Unmodified_shore* − 0.427 x *Per_non_natural*; Multiple-R^2^: 0.389, Adjusted-R^2^ = 0.302, *p* = 0.032; Relative contribution: Per_non_natural (60.1%), Per_Unmodified_shore (39.9%)†
**TP**	**TP** = 2.170*** − **0.581 x** mDepth**; **Multiple R**^**2**^**: 0.470, Adjusted-R**^**2**^ = **0.436, p** = **0.002; Relative contribution: mDepth (100%)**
**Chl-a**	**Chl-a** = 1.178* − **0.846 x** Per_Unmodified_shore* + **0.769 x** ***Per_agri*** + **0.527 x** ***Per_non_natural*** − **0.768 x** mDepth*** + **0.332 x** Wlevel* **Multiple R**^**2**^**: 0.816, Adjusted-R**^**2**^ = **0.739, p** < **0.001; Relative contribution: mDepth (30.9%), Wlevel (21.6%), Per_Unmodified_shore (20.3%), Per_agri (14.5%), Per_non_natural (12.7%)**
**SD**	**SD** = 0.445* + **0.195 x** ***Per_Unmodified_shore*** − **0.420 x** Per_agri* + **0.452 x** mDepth*** − **0.149 x** Wlevel** **Multiple R**^**2**^**: 0.863, Adjusted-R**^**2**^ = **0.820, p** < **0.001; Relative contribution: mDepth (44.3%), Wlevel (24.4%), Per_agri (19.4%), Per_Unmodified_shore (11.9%)**
**pH**	**pH** = −2.764** − **2.582 x** Per_Unmodified_shore** + **2.387 x** Per_agri* + **2.511 x** Per_non_natural** − **1.210 x** ***Per_macro_veg*** + **1.092 x** Wlevel** **Multiple R**^**2**^**: 0.710, Adjusted-R**^**2**^ = **0.589, p** = **0.006; Relative contribution: Wlevel (25.1%), Per_Unmodified_shore (24.5%), Per_non_natural (23.2%), Per_agri (18.4%), Per_macro_veg (8.9%)**
**DO**	**DO** = 1.071*** + **0.048 x** ***Per_Unmodified_shore*** − **0.099 x** Per_agri* − **0.054 x** ***Per_non_natural*** + **0.110 x** Per_macro_veg* − **0.061 x** Wlevel**; **Multiple R**^**2**^**: 0.619, Adjusted-R**^**2**^ = **0.460, p** = **0.03; Relative contribution: Wlevel (35.7%), Per_macro_veg (20.7%), Per_agri (19.4%), Per_non_natural (12.7%), Per_Unmodified_shore (11.5%)**
**NO**_**3**_	NO₃ = −0.211 + 0.632 x Dmax_macro − 0.273 x Per_agri − 0.112 x Wlevel.; Multiple R^2^: 0.292, Adjusted-R^2^ = 0.14, *p* = 0.172; Relative contribution: Wlevel (40.7%), Per_agri (31.8%), Dmax_macro (27.4%)
	**Reservoirs**
**Phyto**	**Phyto** = 0.255*** + **0.020 x** ***Wlevel*** + **0.023 x** ***mDepth_area*** + **0.115 x** ***Nb_natural_100*** − **0.081 x** Per_Int_Agri_100** + **1.464 x** ***Per_Area_basin*** − **0.122 x** Per_Forest* − **0.054 x** ***Per_shrubs*** − **0.251 x** ***Per_low_veg*** **Multiple R**^**2**^**: 0.775, Adjusted-R**^**2**^ = **0.551, p** = **0.049; Relative contribution: Per_Int_Agri_100 (20.6%), Per_Forest (19.2%), Per_Area_basin (13.5%), Per_low_veg (11.3%), Nb_natural_100 (10.5%), mDepth_area (7.7%), Wlevel (7.1%), Per_shrubs (10.1%)**
**TP**	**TP** = 1.660*** − **0.156 x** mDepth** − **0.048 x** mDepth_area** − **0.044 x** Per_Int_Agri_100* − **2.512 x** Per_Area_basin* − **0.080 x** Per_Fallow** **Multiple R**^**2**^**: 0.676, Adjusted-R**^**2**^ = **0.529, p** = **0.00172; Relative contribution: Per_Fallow (25.3%), mDepth_area (20.7%), mDepth (19.9%), Per_Area_basin (18.2%), Per_Int_Agri_100 (15.9%)**
**Chl-a**	**Chl-a** = 1.529*** − **0.323 x** Wlevel* + **0.314 x** Per_Int_Agri_100* − **0.500 x** Per_Fallow** − **0.209 x** ***Per_shrubs*** **Multiple R**^**2**^**: 0.7497, Adjusted-R**^**2**^ = **0.666, p** = **0.001; Relative contribution: Per_Fallow (37.6%), Per_Int_Agri_100 (25.2%), Wlevel (24.8%), Per_shrubs (12.5%)**
**SD**	**SD** = 0.724* − **0.462 x** ***mDepth*** + **0.112 x** ***mDepth_area*** + **0.876 x** Nb_natural_100*** − **5.183 x** ***Per_Area_basin*** + **0.099 x** ***Per_Fallow*** + **0.203 x** ***Per_shrubs*** − **1.046 x** Per_low_veg* **Multiple-R**^**2**^**: 0.873, Adjusted-R**^**2**^ = **0.774, p** = **0.002; Relative contribution: Nb_natural_100 (34.2%), Per_low_veg (13.9%), mDepth (13.0%), mDepth_area (11.0%), Per_shrubs (10.8%), Per_Area_basin (9.3%), Per_Fallow (7.8%)**
**pH**	**pH** = 0.926*** − **0.008 x** ***Retention*** + **0.007 x** ***Wlevel*** + **0.026 x** Per_Int_Agri_100* + **0.345 x** ***Per_Area_basin*** − **0.007 x** ***Per_Fallow*** + **0.015 x** ***Per_Fore*** + **0.009 x** ***Per_shrubs*** + **0.082 x** Per_low_veg* **Multiple-R**^**2**^**: 0.902, Adjusted-R**^**2**^ = **0.803, p** = **0.003; Relative contribution: Per_low_veg (20.7%), Per_Int_Agri_100 (17.4%), Per_Area_basin (15.1%), Wlevel (14.4%), Per_Fore (8.8%), Per_shrubs (8.5%), Retention (7.8%), Per_Fallow (7.2%)**
**DO**	**DO** = 0.948*** − **0.087 x** Retention* + **0.019 x** ***Wlevel*** − **0.052 x** mDepth_area** + **0.045 x** ***Per_Int_Agri_100*** + **0.804 x** ***Per_Area_basin*** + **0.040 x** ***Per_Fore*** − **0.244 x** ***Per_low_veg*** **Multiple-R**^**2**^**: 0.742, Adjusted-R**^**2**^ = **0.541, p** = **0.004; Relative contribution: mDepth_area (28.3%), Retention (18.2%), Per_low_veg (14.0%), Per_Int_Agri_100 (13.3%), Per_Fore (10.7%), Wlevel (7.8%), Per_Area_basin (7.6%)**
**ΝΟ**_**3**_	NO_3_ = 0.677* + 0.203 x Retention** – 0.212 x Wlevel*** − 0.264 x Nb_natural_100* + 0.220 x Per_Fore*** + 0.210 x Per_shrubs*** Multiple-R^2^: 0.796, Adjusted-R^2^ = 0.704, *p* = 0.002; Relative contribution: Wlevel (28.9%), Per_Fore (25.2%), Retention (16.7%), Per_shrubs (15.4%), Nb_natural_100 (13.8%)†

Dmax_macro: Maximum colonization depth of macrophytes; Per_Unmod_shore: complexity and modification percentage of the shoreline, anthropogenic use of littoral areas—unmodified; Per_agri: percentage of arable land, Per_non_natural: percentage of non-natural land cover; Per_macro_veg: coverage of macrophytes in the littoral zone (%); mDepth: mean depth; Wlevel: absolute elevation of water level; mDepth_area: mean depth/lake area; Nb_natural_100: number of natural land cover types; Per_Area_basin: lake area/catchment area; Per_Int_Agri_100: percentage of intensive agriculture; Per_Fallow: percentage of fallow land; Per_Fore: percentage of forests; Per_shrubs: percentage of shrubs and/or herbaceous vegetation; Per_low_veg: percentage of open spaces with little or no vegetation; Phyto: phytoplankton; Macro: macrophytes; TP: total phosphorus (µg L^–1^); Chl-a: chlorophyll-a (µg L^–1^); SD: Secchi Depth (m); pH: acidity; DO: dissolved oxygen (mg L^-1^) and NO_3_: nitrate ([mg L^–1^]). Multiple linear regressions, which are statistically significant and meet the criteria are highlighted in bold. Models highlighted in bold are statistically significant and satisfy the assumptions of linear regression. †: Assumptions for the linear model were not fully satisfied. Significance codes: *** *p* < 0.001; ** *p* < 0.01; * *p* < 0.05; *p* < 0.1

In reservoirs, land use metrics were the dominant (i.e., plurality) predictors of water quality, with morphological and hydrological variables providing additional explanatory power in several models (Table 4). For reservoirs, contributions were more evenly distributed, with no single variable dominating any model (maximum individual contributions ranging from 20.6% to 37.6%), indicating that water quality in reservoirs is shaped by a broader combination of landscape and hydromorphological factors.

### Analysis of HyMo Metrics and Determination of Dominant Factors

**Fig. 4:**
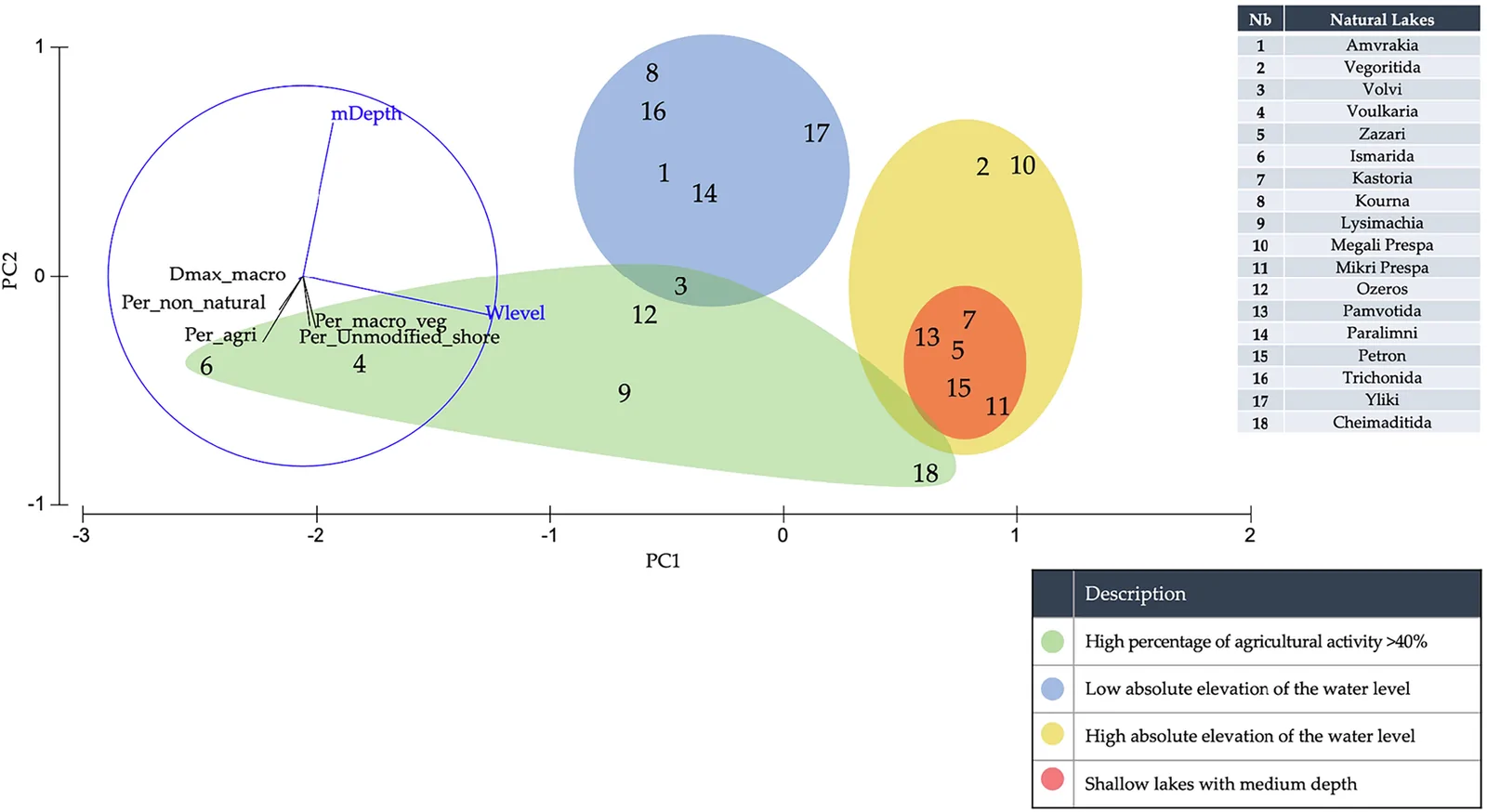
Principal component analysis of hydromorphological metrics in natural lakes. Maximum colonization depth of aquatic macrophytes (Dmax_macro), percentage of shoreline complexity and modification- anthropogenic land use of riparian areas—unmodified (Per_Unmod_shore), percentage of arable land (Per_agri), percentage of non-natural land cover (Per_non_natural), percentage coverage of aquatic vegetation in the overall vegetated coastal zone (Per_macro_veg), mean depth (mDepth), absolute elevation of the water level (Wlevel)

**Fig. 5:**
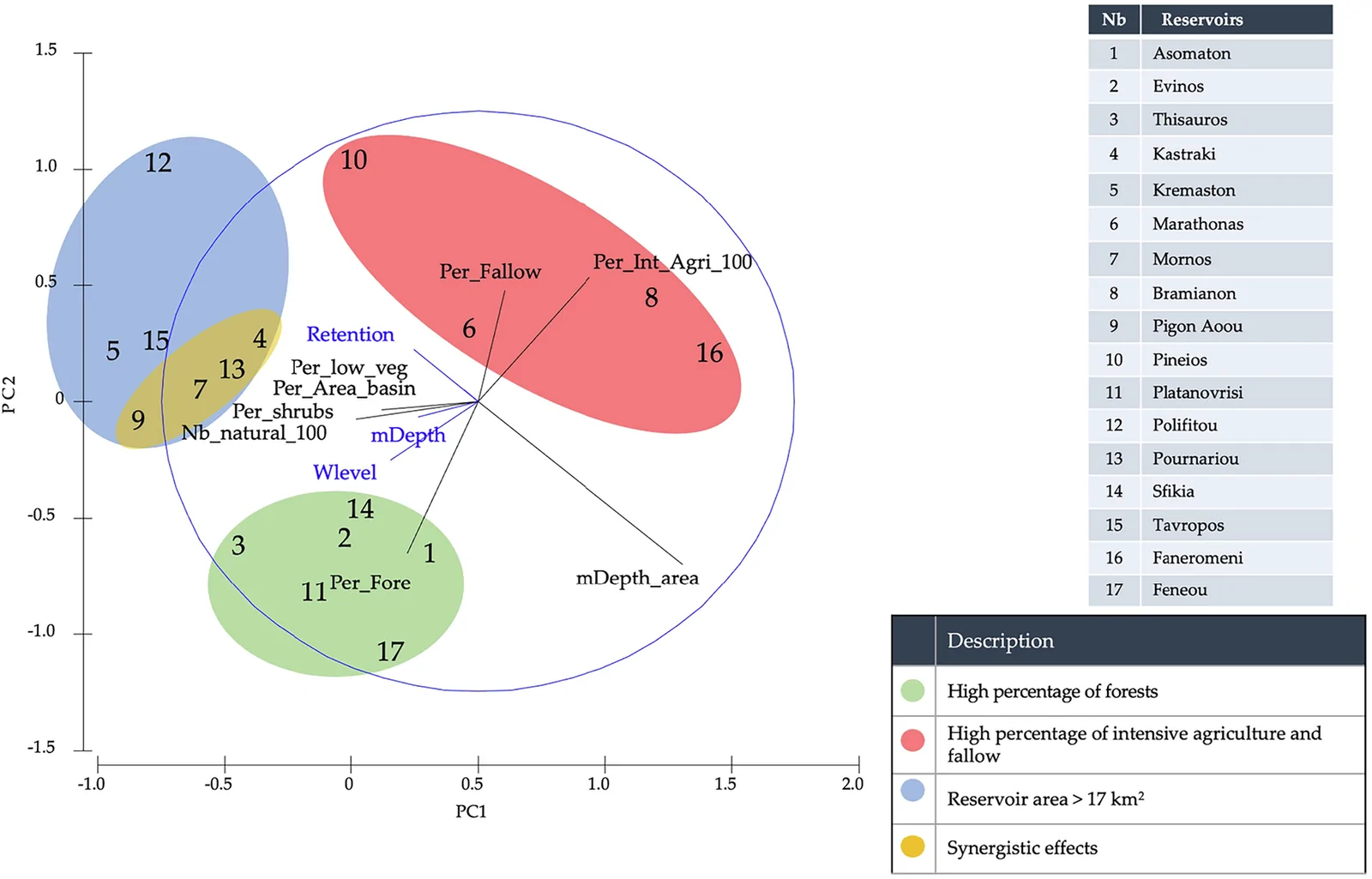
Principal component analysis of hydromorphological metrics in reservoirs. Mean depth (mDepth), retention time (Retention), absolute elevation of the water level (Wlevel), ratio of mean depth to area (mDepth_area), number of natural land cover types (Nb_natural_100), percentage of intensive agriculture (Per_Int_Agri_100), ratio of lake surface area to watershed surface area (Per_Area_basin), percentage of land cover by fallow areas (Per_Fallow), percentage of land cover by forests (Per_Fore), percentage of land cover by shrublands and/or woody vegetation (Per_shrubs), and percentage of land cover characterized by open spaces with little or no vegetation (Per_low_veg)

The PCA of HyMo metrics revealed that the first two axes explained 84.7% of the total variance in natural lakes (PC1: 67.8%, PC2: 16.9%; Fig. 4), while for reservoirs the explained variance was lower (PC1: 38.8%, PC2: 30.5%; Fig. 5). For natural lakes, the axes indicated positive correlations with water level (PC1) and mean depth (PC2) attributes and negatively with agricultural practices (arable land, PC1) and shoreline naturalness (unmodified shoreline complexity, PC2) (Figs. 4 and 5). The analysis identified four distinct lake groups based on their position along the PCA axes, interpreted descriptively to facilitate ecological understanding of the dominant HyMo gradients. The first group (green color, Fig. 4) was characterized by a high percentage of arable land in the buffer zone of 100 m (>40%). The second group (blue, Fig. 4) consisted of low-elevation lakes, distinguished primarily by their absolute water elevation along PC1. The third group (red, Fig. 4) comprised shallow and very shallow lakes with medium depth. The fourth group (yellow, Fig. 4) was characterised by high-elevation lakes, distinguished from the other groups by the combination of mean depth and water level attributes along PC2.

Regarding reservoirs, the ratio of mean depth/area (mDepth_area) was correlated with PC axes (positively PC1 and negatively PC2). PC1 was also negatively affected by the number of natural land cover types (Nb_natural_100) and PC2 by the percentage of land cover by forests (Per_Fore). Similarly, four groups were formed (Fig. 5): (a) represented by a high percentage of land cover by forests (green color), (b) defined by high percentages of intensive agriculture and fallow land (red color), (c) characterized by relatively low ratio of mean depth to lake area (blue color) and (d) reflected synergistic effects of multiple HyMo metrics. (yellow color).

## Discussion

Attempting to respond to the urgent call for developing new methods for the quantitative evaluation of lake HyMo conditions (Poikane 2020), this research manages to showcase interlinkages that describe the way HyMo metrics affect physicochemical and biological QEs, governing the ecological quality status of lakes and reservoirs, as reflected by the EQRs values. While advancing in this field (Argillier et al. 2025), we confront challenges and limitations inherent to our dataset of a limited number of lake water bodies, akin to those encountered by other European and especially Mediterranean (bordering also) countries (Argillier and Carriere 2023). Indeed, several of the selected hydromorphological metrics (e.g., mean depth or the ratio of lake surface area to catchment area) represent not only alterations from natural HyMo conditions but above all, fundamental landscape and hydromorphological characteristics.

### Data Processing, Limitations and Implications for Water Monitoring

The data-mining process was challenging because developing an appropriate indicator set required long-term, consistently monitored data—something largely unavailable for Greek lakes and reservoirs, especially given the dataset’s diversity (with some metrics showing temporal trends and others being single values). Although the dataset covers many HyMo aspects, it cannot capture short-term temporal variability such as hydropeaking (Carriere et al. 2024). Moreover, assigning lakes and reservoirs to clear typological categories remains difficult at the pan-European level (Argillier et al. 2022, 2025).

As for the spatial resolution, CLC 2018 was used in accordance with the national monitoring authority and for this reason the 100 m buffer area was applied. It is acknowledged that the trend in land use change cannot be reflected and the assessment was done according to current (most recent) CLC depiction. A further constraint concerns the spatial resolution of CLC 2018 relative to the scale at which it was applied. The dataset’s minimum mapping unit (25 ha for areal features, and a minimum width of 100 m for linear features; CLC 2018) is comparable to, or in places larger than, the 100-m buffer zone itself, meaning that narrow shoreline habitat patches, small wetland pockets, and riparian vegetation strips falling below this threshold may go undetected or be subsumed into adjacent, coarser land-use classes. Consequently, the land-use metrics derived within these buffers may underrepresent fine-scale shoreline heterogeneity. Higher-resolution products such as the CLCplus Backbone or Sentinel-derived land-cover layers could help resolve these finer-scale patterns in future work, once dataset and computational constraints allow their application across the full set of water bodies. Considerable effort focused on identifying major distortions, including landscape-related pressures. Shoreline naturalness emerged as a key metric for natural lakes, correlating with trophic status. Among hydrological metrics, ecological water level would be valuable for assessing HyMo alterations, but such data exist for only five Greek natural lakes (Petriki et al. 2020; Doulgeris et al. 2024). Other typology-related metrics, such as permeability, mixing/stratification, and climate, were included but difficult to use due to their qualitative nature. A further limitation concerns the potential overlap between HyMo predictors and the macrophyte EQR response variable. Although macrophyte-related metrics (Dmax_macro and Per_macro_veg) were removed from the predictor set to avoid circularity, the HeLM index incorporates maximum colonization depth as a component, representing a residual source of overlap that cannot be fully eliminated given the pre-existing structure of the national index (Zervas et al. 2018).

Metrics related to hydrological, sedimentary, and landscape connectivity (Taylor et al. 2025) were prioritized because they strongly affect the ecological continuum (Argillier et al. 2022, 2025). These included drainage, hydraulic infrastructure, flood-protection works, port facilities, controlled hydrology, and proximity to wetlands. Several habitat-based metrics were also considered, though not all were statistically significant. Land use within a 100-m buffer proved particularly influential, affecting both biological and physicochemical QEs. Fallow land, natural vegetation, and intensive agriculture were especially important, as confirmed by the study’s results.

A further limitation concerns the ratio of explanatory variables to water bodies retained through forward selection, which is below the commonly recommended guideline of approximately 10 observations per predictor (Harrell 2015). Such ratios increase the risk of model overfitting, inflated explained variance, and unstable parameter estimates, thereby reducing model robustness. Nevertheless, the final set of predictors was not selected a priori but retained through forward selection after screening for multicollinearity, ensuring that only variables providing a statistically significant and ecologically meaningful contribution to the ordination were included. Consequently, the identified environmental gradients should be regarded as exploratory, consistent with similar limitations reported for national scale hydromorphological datasets (Argillier and Carriere 2023).

In summary, the current analyses suggest that monitoring of HyMo parameters could be strengthened by increasing the frequency of hydrological measurements and incorporating remote sensing technologies (e.g., Perivolioti et al. 2025). For Greece, metrics from the LHS and Lake-MImAS (Rowan et al. 2012), though demanding, could offer valuable complementary information beyond the current dataset, particularly regarding lake functionality, geospatial diversity, and disturbances identifiable through on-site observations.

### Environmental Gradient Analysis

The RDA revealed associations between HyMo metrics and biological and physicochemical QEs, identifying gradients of positive and negative relationships. Regarding natural lakes, benthic macroinvertebrates were associated with the percentage of unmodified shoreline and water level, which is expected as the BQE index targets the man-affected littoral habitats (Mavromati et al. 2023). Both phytoplankton and macrophytes were positively associated with mean depth, suggesting greater resilience in deeper aquatic systems, while negative associations were observed with agriculture-related metrics and non-natural land cover. Mean depth expressing the tolerance and self-purification ability of natural lake systems against eutrophication stressors (Genkai-Kato and Carpenter 2005, Jäger et al. 2008) can also be witnessed by its correlations with shoreline complexity and modification, the extent of unmodified littoral areas, maximum colonization depth of aquatic macrophytes and concentrations of Chl-a and nutrients. Eutrophication metrics describe better EQR as quality descriptors and hence HyMo metrics associated with eutrophication/purification effects (Kutyła et al., 2025) or landscape/lakeshore (Peterlin and Urbanič 2013) naturalness may create “stronger links”.

In reservoirs, the RDA recognized that reduced pressure from agriculture, i.e., fallow periods and crop rotation, along with more natural land cover (i.e., shrubs, forest, low vegetation) and lower water residence time was associated with higher EQR values for phytoplankton. This is also reflected in the increase of the euphotic zone, as reflected in transparency and the Secchi depth, while the mean depth of lakes seems to increase the resilience of the systems themselves, as it is negatively correlated with TP and NO_3_ concentrations (Bucak et al. 2018). Also, the connection of areas related to intensive agricultural practices is reflected in increased Chl-a concentrations.

### Influence of Key HyMo Metrics on QEs

In natural lakes, the two BQEs (phytoplankton and macrophytes) showed associations with HyMo metrics, as well as Chl-a and dissolved oxygen. Phytoplankton EQR values were linked to substrate composition, turbidity, shoreline naturalness, water level and land-use metrics (Søndergaard et al. 2013). Macrophyte EQR values showed similar associations, particularly with non-natural land cover and water-level elevation (Cheruvelil and Soranno 2008), likely reflecting interactions with phytoplankton and nutrient availability (Søndergaard et al. 2017). Dissolved oxygen showed relations to land cover pressures (non-natural and agricultural) that deplete it (Crossman et al. 2019), macrophytes and water level. TP was mainly related to mean depth, which as a morphological metric may reflect the natural mixing regime of the water body.

Beyond its well-documented role in directly increasing nutrient inputs through fertilizer runoff, agricultural land cover may exert multiple indirect hydromorphological effects on lake ecosystems. Surface runoff from cultivated fields accelerates sediment delivery, altering substrate composition in littoral zones and smothering macrophyte beds and benthic habitats (Heathcote et al. 2013). Furthermore, agricultural expansion is frequently associated with the loss of natural shoreline and riparian vegetation, which plays a critical role in bank stabilisation, diffuse pollutant filtering, and maintaining the structural complexity of littoral habitats (Brauns et al. 2007; Porst et al. 2019). This is consistent with the negative associations observed between Per_non_natural land cover and benthic macroinvertebrate EQR values in our dataset. These findings suggest that agricultural land cover may act as a compound stressor, affecting lake ecosystems through multiple hydromorphological pathways beyond nutrient enrichment alone.

In reservoirs, six QEs (phytoplankton, Chl-a, TP, Secchi depth, pH and dissolved oxygen) correlated significantly with HyMo metrics. Phytoplankton EQR values were associated with eight hydrological and morphological metrics across all HyMo pressures, reflecting its short life cycle and sensitivity to nutrients, light, and hydrology (Kruk et al. 2017). Mean depth, a proxy for dilution capacity and resilience (Jäger et al. 2008), along with its ratio to lake area, showed positive associations with phytoplankton EQR values, suggesting a potential link with greater buffering capacity in deeper systems. Natural land cover metrics (i.e., shrubs, forest, low vegetation, and number of natural types) were negatively associated with intensive agriculture and positively with phytoplankton EQR values (Katsiapi et al. 2012). Chl-a concentration was linked to pressures from intensive agriculture and land management practices such as fallow land and shrubs, suggesting a potential link with lake productivity (Søndergaard et al. 2017). For TP, associations were observed with mean depth, its ratio to lake area, and the lake area to basin area ratio, along with intensive agriculture and fallow land, indicating that both background morphological descriptors and land use pressure variables may jointly influence phosphorus dynamics. The pH was correlated with water level, retention time, the lake area to basin area ratio, and natural land cover types, consistent with the influence of soil type and composition along with biological activity (Nõges 2009). Secchi depth showed positive associations with metrics suggesting enhanced transparency, such as mean depth, diverse land cover, fallow, shrubs, and low vegetation, and showed a negative association with residence time, in line with the known relationship between water renewal rate and transparency in lake systems (Jeppesen et al. 2007). Dissolved oxygen is similarly related to residence time, the two ratios (i.e., mean depth/lake area and lake area/basin area) indicating dilution capacity, and land uses (intensive agriculture, low vegetation) linked to nutrient inputs.

It is particularly notable that in each case, hydrological and morphological metrics consistently appeared in the correlations with QEs, suggesting their interconnected role in influencing ecological status as captured by the EQR indices. Depending on the linearity of relationships, the identified correlations can be used to quantify the magnitude of influence of HyMO metrics on QEs.

### Dominant HyMo Features According to Lake Typology

A well-dispersed distribution of natural lakes and reservoirs was revealed by PCA and classifying them into meaningful groupings. Particularly for natural lakes, four distinct groups were formed, associated with typological descriptors, such as mean depth and absolute elevation, alongside land use variables and shoreline modification (i.e., agricultural activity and shoreline complexity). Notably, it grouped lakes with a percentage of arable land greater than 40%, which is considered an important pressure (Sexton et al. 2024). On the contrary, the results for reservoirs showed quite different ecological dynamics. The relatively reduced influence from shoreline morphology and the natural composition of habitats was associated with a shift in the emphasis towards hydrological characteristics and the magnitude of pressures exerted by the catchment basin (Wang et al. 2022). Consequently, PCA was primarily structured by the ratio of mean depth to lake area, followed by the number of different natural land cover types. The groups shaped by morphological parameters, land use patterns and agricultural practices (i.e., forested areas versus agricultural zones), are indicative of direct environmental pressures. Generally, various environmental attributes, such as buffer zones, anthropogenic activities, and different trophic states affecting physicochemical parameters, tend to exhibit stronger and more consistent associations than those observed for HyMo metrics (Kutyła et al. 2024).

### Implications for the WFD Assessment of HyMo QEs

Following the above analyses and consistent with Argillier and Carriere (2023), metrics under the broader category of “Lake shore structure” were identified, particularly those describing physical characteristics such as the proportion of unmodified shoreline. Metrics linked to “Quantity and dynamics of water flow” were also classified, including absolute water-level elevation and water residence time. Considerable effort was made to develop indicators related to impoundment in the catchment (e.g., artificial structures), though these showed no strong correlations with biotic or abiotic factors. Likewise, metrics on lake–groundwater connectivity (“Connection with groundwater”) were examined, but statistical analysis was not feasible. Another category initially lacking data was “Lake Use”, which is common in Europe but limited in Greece, where uses mainly involve fishing, irrigation water extraction, flood protection, and tourism, rather than navigation, aquaculture, or energy production noted elsewhere in the EU over a decade ago (Poikane et al. 2015). Conversely, several important HyMo metrics (e.g., wetland extent, non-natural land cover within 100 m, intensive agriculture coverage) were derived from existing data, highlighting land-use patterns in Greek and Mediterranean basins, where agriculture is a key driver. In this context, alternative indicators of substrate naturalness, such as submerged macrophyte colonization depth or hydrophyte prevalence in riparian zones, can substitute for direct references to the “lakebed” in Greek natural lakes. It comes with agreement with the notion of Poikane et al. (2020) that hydromorphological pressures and their effects should be reflected in HyMo assessment methods and should be properly monitored alongside the biological methods.

### Implications for Ecohydrological Management Strategies

Lake hydrological regulation is widespread and recognized as a major pressure in assessment methods (Kampa et al. 2019) and in recent EU initiatives, including the Nature Restoration Regulation and the Biodiversity Strategy. Although no strong correlations emerged in our dataset, its ecological importance warrants further investigation, especially regarding impacts on aquatic biota.

In Greece, the HyMo patterns identified here are potentially relevant at the national level and may contribute to efforts to address long-standing gaps in lake and reservoir assessment. The current national system remains restrictive due to few incorporated metrics, highlighting the need for more detailed primary data. Still, the progress observed is broadly consistent with broader European developments. Within the RBMPs, metrics such as the “Proportion of perimeter under intensive land use (B.4.1)” and the “Percentage modified by embankments (B.2.1)” may represent promising candidate tools for improved HyMo evaluation.

Our findings indicate that a future index could potentially integrate multiple metrics while distinguishing natural lakes from reservoirs, though formal index development would require larger datasets. Chl-a concentrations showed associations with shoreline type, agriculture, and depth for natural lakes, whereas land use categories and water level seemed to be more decisive factors for Greek reservoirs. Lake type specific research could also take place exploiting further spatial analysis using more detailed land cover data e.g., CLCplus Backbone 2023, which could not be applied in this study due to the constraints of the dataset and the number of water bodies. Likewise, Secchi depth showed associations with agricultural practices and basic hydrological features for both reservoirs and natural lakes, with the latter being further affected by shoreline modifications (Søndergaard et al. 2007; Nielsen et al. 2012). Overall, these patterns underscore the value of expanding hydrological datasets and adopting more nuanced, system-specific approaches to ecohydrological management across Greek lake ecosystems.

## Conclusions

Although links between hydrological and morphological parameters across spatial units (e.g., shores, buffer zones, catchments) were known before the WFD, systematic scientific work and coordinated assessment methods advanced only after its implementation, yielding important results (Ciampittiello et al. 2017). This study is the first to explore and quantify associations among BQEs, abiotic parameters and HyMo metrics for Greek lakes and reservoirs. Based on ecohydrological principles, it identifies a set of candidate metrics that may inform future monitoring frameworks under the WFD, though further validation would be required before these can be considered definitive monitoring priorities.

A comprehensive, regularly updated monitoring dataset is crucial for national compliance. To address current gaps, we developed a set of recombined metrics capturing a wide range of HyMo characteristics. These metrics, representing both anthropogenic pressures and typological factors, effectively characterize Greek lakes by suggesting major gradients associated with lake differentiation and, in some cases, patterns potentially linked to ecosystem functioning. Ecological quality in Greek lakes and reservoirs seems to respond to distinct combinations of HyMo and landscape metrics. In natural lakes, BQEs respond mainly to shoreline naturalness, depth, water level, and agricultural land use, suggesting links with littoral and deep-water dynamics. Reservoirs showed stronger associations with hydrological regimes, land cover, and agricultural activity, with metrics such as mean depth, residence time, and lake-to-basin area ratios most consistently related to ecological patterns. Across both system types, lake typology and dominant HyMo pressures produce groupings, underscoring the potential value of integrated, type-specific ecohydrological assessment and management. BQEs differ in their sensitivity to HyMo metrics, and similar variability appears in parameters such as transparency, Chl-a, and TP. Regarding nitrates and pH, their relationships with HyMo metrics were generally weak in natural lakes; however, pH in reservoirs showed relatively stronger associations with hydrological and morphological variables. The broad set of candidates HyMo metrics (Argillier et al. 2025) expands the pool of potentially useful indicators for effective ecohydrological management of lakes.

## Supplementary information

Supplementary material

## Data Availability

Data sets generated during the current study are available from the corresponding author on reasonable request.
