# Supplementary material for: How Hydromorphological Pressures Co-govern Lake Water Quality in a Mediterranean Environment. A Study for Greek Natural Lakes and Reservoirs
Source: Environ Manage. 2026 Sep 17;76(10):324. doi: 10.1007/s00267-026-02627-6 (PMC13585854; doi:10.1007/s00267-026-02627-6)
Supplement: Supplementary file 1 [file 267_2026_2627_MOESM1_ESM.docx]

**Supplementary material**

**Table S1.** Hydromorphological features of Greek natural lakes and reservoirs. Values are presented as mean ± standard deviation (minimum – maximum) based on annual data for the period 2012–2021. Lake perimeter and catchment area are presented as single values. Absolute elevation refers to the water surface level above mean sea level (amsl). (–) indicates unavailable data.

| **Water body** | **Latitude (°N)** | **Longitude (°E)** | **Lake area (km^2^)** | **Lake perimeter (km^2^)** | **Mean depth (m)** | **Volume (m^3^)** | **Retention time (yr)** | **Absolute elevation of water level (amsl)** | **Catchment area (km^2^)** |
| --- | --- | --- | --- | --- | --- | --- | --- | --- | --- |
| **Natural lakes** | | | | | | | | | |
| Amvrakia | 38.752 | 21.178 | 12.11±1.09  (10.51 - 13.25) | 19.44 | 24.09±0.73  (23.29 - 25.02) | 285.60±12.97  (265.33 - 298.94) | 12.50 | 18.94±1.03  (16.86 - 19.72) | 121.10 |
| Vegoritis | 40.750 | 21.783 | 47.25±0.45  (46.53 - 47.67) | 42.93 | 26.03±0.47  (25.33 - 26.52) | 1212.66±29.94  (1164.48- 1240.78) | 9.00 | 517.08±1.35  (517.11 - 518.73) | 1791.6 |
| Volvi | 40.682 | 23.467 | 71.74±1.90  (69.85 - 74.94) | 57.60 | 12.38±0.14  (12.17 - 12.54) | 892.65±39.94  (849.69 - 959.58) | 8.40 | 35.73±0.50  (35.14 - 36.06) | 1282.70 |
| Voulkaria | 38.866 | 20.841 | 7.10±1.02  (5.27 - 7.91) | 17.41 | 0.94±0.03  (0.87 - 0.96) | 6.69±1.14  (4.59 - 7.54) | 2.00 | 1.80±0.15  (1.49 - 1.95) | 74.40 |
| Doirani | 41.220 | 22.750 | 32.66±0.38  (32.18 - 33.25) | 19.71 | 4.58±0.04  (4.52 - 4.64) | 149.49±3.24  (145.42 - 154.58) | - | 145.89±1.06  (144.06 - 146.78) | 339.70 |
| Distos | 38.340 | 24.116 | - | 11.27 | - | - | - | 13.17±0.35  (12.74 - 13.83) | 57.50 |
| Zazari | 40.625 | 21.547 | 1.94±0.02  (1.91 - 1.97) | 6.87 | 4.04±1.48  (1.01 - 4.72) | 9.04±0.26  (8.71 - 9.35) | 1.57 | 599.32±0.20  (598.86 - 599.52) | 99.30 |
| Ismarida | 40.984 | 25.318 | 1.76±0.14  (1.61 - 2.01) | 13.94 | 0.81±0.06  (0.75 - 0.90) | 1.44±0.22  (1.23 - 1.81) | - | 0.32±0.08  (0.25 - 0.42) | 358.70 |
| Kastoria | 40.515 | 21.300 | 30.51±0.34  (30.22 - 31.01) | 34.15 | 3.68±0.03  (3.65 - 3.72) | 112.36±2.17  (110.33 - 115.52) | 3.40 | 626.95±0.08  (626.83 - 627.08) | 315.00 |
| Koronia | 40.683 | 23.150 | - | 31.46 | - | - | - | 70.28±0.74  (69.26 - 71.38) | 781.40 |
| Kourna | 35.331 | 24.276 | 0.5 | 3.36 | - | - | - | 16.46±0.85  (14.81 - 15.68) | 10.80 |
| Lysimachia | 38.561 | 21.369 | 10.60±0.17  (10.40 - 10.87) | 22.38 | 3.27±0.18  (3.04 - 3.52) | 34.64±2.47  (31.58 - 38.27) | 1.00 | 15.36±0.26  (14.81 - 15.68) | 254.30 |
| Megali Prespa | 40.900 | 21.033 | 274.00 | 39.34 | 17.00 | 4800.00 | - | 842.55 | 1129.30 |
| Mikri Prespa | 40.767 | 21.100 | 45.17±2.02  (43.74 - 46.60) | 50.01 | 3.96±0.01  (3.95 - 3.97) | 178.92±7.37  (173.71 - 184.13) | 2.17 | 849.60 | 265.90 |
| Ozeros | 38.656 | 21.224 | 10.48±0.08  (10.40 - 10.59) | 13.54 | 3.73±0.09  (3.63 - 3.83) | 38.91±1.19  (37.70 - 40.52) | 4.20 | 24.14±0.09  (24.03 - 24.30) | 57.40 |
| Pamvotida | 39.663 | 20.885 | 21.77±0.12  (21.64 - 21.97) | 27.06 | 5.25±0.11  (5.13 - 5.45) | 114.20±3.07  (110.94 - 119.66) | 2.00 | 468.92±0.15  (468.69 - 469.09) | 528.80 |
| Paralimni | 38.463 | 23.360 | 12.13±2.12  (9.96 - 14.51) | 18.07 | 8.49±4.82  (2.99 - 13.86) | 113.63±74.32  (42.56 - 201.15) | 5.90 | 40.31±5.26  (35.77 - 48.43) | 74.50 |
| Petron | 40.727 | 21.699 | 11.72±0.19  (11.53 - 11.97) | 18.14 | 2.96±0.13  (2.83 - 3.14) | 33.89±2.02  (31.84 - 36.54) | 1.70 | 572.57±0.17  (572.36 - 572.81) | 219.20 |
| Pikrolimni | 40.835 | 22.813 | 5.45±1.20  (3.99 - 6.67) | 11.02 | 1.09±0.63  (0.40 - 2.07) | 6.52±4.77  (1.60 - 13.79) | 4.20 | 44.54±0.88  (43.63 - 45.80) | 40.60 |
| Saltini | 38.917 | 20.767 | - | 9.15 | - | - | - | - | 13.00 |
| Stimfalia | 37.851 | 22.461 | - | 10.23 | - | - | - | - | 216.40 |
| Trichonida | 38.573 | 21.553 | 93.39±0.66  (92.77 - 94.63) | 52.53 | 29.33±0.25 (28.88 - 29.61) | 2739.13±19.19  (2,712.51 - 2,769.33) | 35.10 | 16.17±0.19  (15.82 - 16.43) | 401.90 |
| Yliki | 38.400 | 23.267 | 21.73±1.57  (19.74 - 23.21) | 54.41 | 21.65±1.58  (19.89 - 23.43) | 472.43±67.47  (392.59 - 543.83) | 2.35 | 74.27±2.43  (70.68 - 77.71) | 2403.30 |
| Cheimaditida | 40.590 | 21.565 | 9.58±0.51  (8.54 - 9.82) | 17.38 | 1.61±1.53  (0.95 - 4.74) | 9.59±0.50  (8.62 - 9.90) | 5.60 | 591.46±0.10  (591.34 - 591.65) | 35.10 |
| **Reservoirs** | | | | | | | | | |
| Asomaton | 40.474 | 22.244 | 2.47±0.02  (2.44 - 2.49) | 23.19 | 20.84±0.17  (20.58 - 21.11) | 51.64±0.73  (50.52 - 52.78) | 1.02±0.11  (0.90 - 1.14) | 84.33±0.29  (83.88 - 84.82) | 67.90 |
| Gratini | 41.150 | 25.533 | 0.77±0.03  (0.74 - 0.81) | 7.11 | 13.97±0.35  (13.60 - 14.52) | 10.83±0.65  (10.06 - 11.72) | - | 136.71±0.68  (135.83 - 137.92) | 68.40 |
| Evinos | 38.667 | 21.864 | 2.90±0.23  (2.68 - 3.28) | 17.61 | 33.34±1.90  (31.54 - 36.36) | 97.07±13.60  (84.53 - 119.39) | 0.50±0.15  (0.35 - 0.76) | 492.65±4.47  (487.85 - 499.67) | 352.90 |
| Thisauros | 41.354 | 24.367 | 12.96±0.95  (11.11 - 13.73) | 37.38 | 38.35±0.08  (38.19 - 38.41) | 502.61±36.01  (432.87 - 533.08) | 0.76±0.23  (0.47 - 1.02) | 366.21±2.36  (360.12 - 368.21) | 4,233.60 |
| Karla | 39.533 | 22.700 | 30.02±0.81  (29.44 - 30.59) | 29.96 | 0.95±0.06  (0.90 - 0.99) | 28.42±2.67  (26.53 - 30.30) | 1.00 | - | 1,662.70 |
| Kastraki | 38.742 | 21.364 | 25.02±0.51  (24.37 - 25.58) | 114.16 | 32.90±0.28  (32.54 - 33.21) | 793.58±5.26  (786.89 - 799.23) | 2.03±0.65  (1.23 - 3.05) | 144.64±0.26  (144.27 - 145.05) | 541.60 |
| Kerkini | 41.210 | 23.130 | 59.40±2.34  (55.77 - 62.31) | 50.17 | 2.55±0.23  (2.19 - 2.83) | 152.72±18.96  (123.10 - 176.12) | 0.09±0.65  (0.08 - 0.09) | 33.36 | 11,599.30 |
| Kremaston | 38.887 | 21.495 | 66.67±3.21  (62.17 - 70.46) | 211.27 | 46.27±1.56  (43.71 - 47.74) | 3,453.78±276.20  (3,076.65 - 3,782.88) | 1.56±0.60  (0.97 - 2.60) | 263.10±3.82  (256.23 - 268.44) | 3,567.80 |
| Ladona | 37.760 | 22.010 | - | 31.24 | - | 31.39±2.25  (29.14 - 35.37) | 0.14±0.04  (0.08 - 0.20) | 414.26±1.03  (412.51 - 415.72) | 767.30 |
| Leukogeion | 41.401 | 23.911 | 0.89±0.15  (0.72 - 1.08) | 7.27 | 5.12±2.94  (2.86 - 8.45) | 4.85±3.76  (2.06 - 9.13) | 0.87±0.61  (0.37 - 1.56) | 608.44±4.01  (602.12 - 614.27) | 53.00 |
| Marathonas | 38.169 | 23.900 | 2.17±0.01  (2.16 - 2.19) | 14.08 | 15.76±0.08  (15.69 - 15.88) | 34.20±0.35  (33.84 - 34.79) | 0.85±0.11  (0.67 - 0.94) | 220.10±0.22  (219.79 - 220.41) | 118.30 |
| Mornos | 38.543 | 22.154 | 17.10±0.59  (16.29 - 17.73) | 65.92 | 37.85±1.04  (36.46 - 39.10) | 647.71±39.96  (593.96 - 693.31) | 1.74±0.54  (1.20 - 2.67) | 428.63±2.31  (425.29 - 431.17) | 584.10 |
| Bramianon | 35.039 | 25.699 | 0.70±0.22  (0.40 - 0.95) | 6.51 | 9.77±2.07  (6.90 - 12.09) | 7.26±3.53  (2.76 - 11.50) | 0.84±0.38  (0.41 - 1.13) | 65.04±4.95  (54.57 - 70.29) | 26.40 |
| Adriani | 41.136 | 24.267 | - | 3.66 | - | 0.95 | 1.00 | 4.28±0.37  (3.57 - 4.77) | 6.60 |
| Pigon Aoou | 39.836 | 21.067 | 11.09±0.54  (10.01 - 11.45) | 46.56 | 20.29±0.77  (18.79 - 20.85) | 225.23±18.86  (188.27 - 238.73) | 2.64±1.11  (1.72 - 4.63) | 1,338.95±2.20  (1,332.93 - 1,340.33) | 87.00 |
| Pineios | 37.896 | 21.485 | 17.31±2.61  (13.80 - 19.90) | 69.39 | 14.39±0.87  (13.25 - 15.30) | 250.45±54.23  (175.77 - 304.53) | 1.06±0.27  (0.69 - 1.30) | 87.01±2.78  (82.41 - 90.52) | 705.20 |
| Platanovrisi | 41.168 | 24.382 | 2.99±0.02  (2.96 - 3.03) | 32.48 | 26.41±0.02  (26.37 - 26.44) | 67.31±0.80  (66.12 - 68.55) | 0.51±0.16  (0.29 - 0.77) | 225.47±0.25  (225.07 - 225.90) | 399.50 |
| Polifitou | 40.302 | 22.101 | 60.85±2.39  (57.03 - 63.49) | 113.60 | 21.56±0.79  (20.27 - 22.41) | 1,540.67±92.60  (1,393.59 - 1,641.56) | 4.58±0.36  (4.22 - 5.15) | 284.93±1.16  (282.40 - 286.45) | 5,518.60 |
| Pournariou | 39.185 | 21.025 | 19.12±0.15  (18.86 - 19.28) | 75.50 | 29.42±0.39  (28.80 - 29.83) | 562.55±11.83  (543.10 - 574.26) | 0.53±0.36  (0.30 - 0.97) | 113.10±0.63  (111.62 - 113.72) | 1,809.70 |
| Pournariou II | 39.150 | 20.990 | 0.57±0.01  (0.55 - 0.59) | 6.30 | 11.79±0.15  (11.66 - 12.07) | 6.70±0.18  (6.50 - 6.93) | 0.01±0.01  (0.00 - 0.02) | 37.98±0.24  (37.74 - 38.50) | 6.50 |
| Smokovo | 39.147 | 22.063 | - | 35.31 | - | - | - | 359.70±5.46  (352.84 - 365.34) | 368.60 |
| Stratos | 38.676 | 21.336 | 7.03±0.01  (7.01 - 7.05) | 26.36 | 9.65±0.06  (9.59 - 9.75) | 71.79±0.57  (71.22 - 72.80) | 0.54 | 67.51±0.08  (67.38 - 67.65) | 225.90 |
| Sfikia | 40.397 | 22.192 | 3.98±0.04  (3.94 - 4.03) | 20.98 | 23.22±0.01  (23.21 - 23.22) | 90.84±1.08  (89.94 - 92.40) | 1.27±0.12  (1.15 - 1.46) | 143.91±0.31  (143.33 - 144.36) | 172.90 |
| Tavropos | 39.236 | 21.746 | 21.12±0.27  (20.88 - 21.46) | 91.03 | 14.83±0.15  (14.70 - 15.02) | 311.64±8.03  (303.50 - 322.29) | 2.50±0.31  (2.03 - 2.85) | 787.92±0.64  (786.34 - 788.61) | 167.00 |
| Faneromeni | 35.099 | 24.853 | 0.56±0.18  (0.33 - 0.76) | 8.53 | 13.64±2.68  (9.98 - 16.49) | 7.98±3.92  (3.31 - 12.55) | 0.74±0.49  (0.21 - 1.33) | 143.30±7.24  (133.72 - 151.17) | 90.90 |
| Feneou | 37.860 | 22.290 | 0.46±0.01  (0.45 - 0.47) | 4.22 | 10.46±0.05  (10.40 - 10.52) | 4.82±0.08  (4.71 - 4.92) | 1.47 | 872.19±0.18  (871.87 - 872.38) | 16.70 |

**Table S2.** Metrics used in the analyses include recombined indices and typological descriptors, grouped into 24 basic morphological, 4 hydrological metrics and 6 typological metrics.

| **Category** | **Metrics** | | | **Abbreviation** | | |
| --- | --- | --- | --- | --- | --- | --- |
| **Morphological parameters** | **1. Lake area (km^2^, EKBY)** | | | **Area** | | |
|  | **2. Lake perimeter (km, EKBY)** | | | **Perimeter** | | |
|  | **3. Mean depth (m, EKBY)** | | | **mDepth** | | |
|  | 3.1. Mean depth deviation | | | mDepth_dev | | |
|  | 3.2. Mean depth deviation (%) | | | Per_mDepth | | |
|  | 3.3. Mean depth/Lake area | | | mDepth_area | | |
|  | 3.4. Mean depth/Lake perimeter | | | mDepth_perimeter | | |
|  | **4. Maximum colonization depth of macrophytes (m; EKBY, 2021)** | | | **Dmax_macro** | | |
|  | **5. Percentage of littoral microhabitats (EEA, 2020)** | | | | | |
|  | 5.1 | Hard littoral microhabitats (2.5m, %) | Per_Hard_2.5 | |  |  |
|  | 5.2 | Soft littoral microhabitats (2.5m, %) | Per_Soft_2.5 | |  |  |
|  | 5.3 | Unmodified littoral microhabitats (2.5m, %) | Per_Unmodified_2.5 | |  |  |
|  | 5.4 | Hard littoral microhabitats (5m, %) | Per_Hard_5 | |  |  |
|  | 5.5 | Soft littoral microhabitats (5m, %) | Per_Soft_5 | |  |  |
|  | 5.6 | Unmodified littoral microhabitats (5m, %) | Per_Unmodified_5 | |  |  |
|  | 5.7 | Hard littoral microhabitats (15m, %) | Per_Hard_15 | |  |  |
|  | 5.8 | Soft littoral microhabitats (15m, %) | Per_Soft_15 | |  |  |
|  | 5.9 | Unmodified littoral microhabitats (15m, %) | Per_Unmodified_15 | |  |  |
|  | 5.10 | Hard littoral microhabitats (-2.5m, %) | Per_Hard_m2.5 | |  |  |
|  | 5.11 | Soft littoral microhabitats (-2.5m, %) | Per_Soft_m2.5 | |  |  |
|  | 5.12 | Unmodified littoral microhabitats (-2.5m, %) | Per_Unmodified_m2.5 | |  |  |
|  | 5.13 | Hard littoral microhabitats (-5m, %) | Per_Hard_m5 | |  |  |
|  | 5.14 | Soft littoral microhabitats (-5m, %) | Per_Soft_m5 | |  |  |
|  | 5.15 | Unmodified littoral microhabitats (-5m, %) | Per_Unmodified_m5 | |  |  |
|  | 5.16 | Hard littoral microhabitats (-15m, %) | Per_Hard_m15 | |  |  |
|  | 5.17 | Soft littoral microhabitats (-15m, %) | Per_Soft_m15 | |  |  |
|  | 5.18 | Unmodified littoral microhabitats (-15m, %) | Per_Unmodified_m15 | |  |  |
|  | **6. Percentage of different littoral habitats based on substrate category (EEA, 2020)** | | | | | |
|  | 6.1 | Macrophytes at 2.5m around lake (%) | Per_Macro_2.5 | |  |  |
|  | 6.2 | Rocks at 2.5m around the lake (%) | Per_rocks_2.5 | |  |  |
|  | 6.3 | Sand at 2.5m around the lake (%) | Per_sand_2.5 | |  |  |
|  | **7. Macrophytes as a function of distance from shoreline (-15m) and substrate composition (EEA, 2020)** | | | | | |
|  | 7.1 | Number of macrophytes as a function of distance from shoreline (-15m) and substrate composition | Nb_macro_m15 | |  |  |
|  | 7.2 | Area of macrophytes as a function of distance from shoreline (-15m) and substrate composition | Area_macro_m15 | |  |  |
|  | **8. Number of recorded natural land cover types (CLC, 2018)** | | | | | |
|  | 8.1 | Number of recorded natural land cover types (50m, categories 3.1.1, 3.1.2, 3.1.3, 3.2, 3.3, 4 & 5) | Nb_natural_50 | |  |  |
|  | 8.2 | Number of recorded natural land cover types (100m, categories 3.1.1, 3.1.2, 3.1.3, 3.2, 3.3, 4 & 5) | Nb_natural_100 | |  |  |
|  | **9. Percentage of intensive agriculture area (CLC, 2018)** | | | | | |
|  | 9.1 | Intensive Agriculture (50m, %, categories 2.1, 2.2, 2.4.1, 2.4.2) | Per_Int_Agri_50 | |  |  |
|  | 9.2 | Intensive Agriculture (100m, %, categories 2.1, 2.2, 2.4.1, 2.4.2) | Per_Int_Agri_100 | |  |  |
|  | **10. Population density (number of inhabitants/km^2^)** | | | **Pop_dens** | | |
|  | **11. Percentage of shoreline complexity and modification, anthropogenic use of littoral areas (Mavromati et al., 2023)** | | | | | |
|  | 11.1 | Hard (%) | Per_Hard_shore | |  |  |
|  | 11.2 | Soft (%) | Per_Soft_shore | |  |  |
|  | 11.3 | Unmodified (%) | Per_Unmod_shore | |  |  |
|  | **12. Presence of hydrotechnical structures (RBMPs, 2023)** | | | | | |
|  | 12.1 | In outflow | Output | |  |  |
|  | 12.2 | In inflow | Input | |  |  |
|  | **13. Shoreline with anthropogenic development (Mavromati et al., 2023)** | | | | | |
|  | 13.1 Shoreline with anthropogenic development (m) | | | Shore_artificial | | |
|  | 13.2 Shoreline with anthropogenic development (%) | | | Per_Shore_artificial | | |
|  | **14. Land cover in shoreline (Mavromati et al., 2023)** | | | | | |
|  | 14.1 | Sand (m) | Sand | |  |  |
|  | 14.2 | Rocks (m) | Rock | |  |  |
|  | 14.3 | Wetland seasonally flooded (m) | wet_meadow | |  |  |
|  | 14.4 | Natural categories (m) | Natural_cat | |  |  |
|  | 14.5 | Agricultural areas (m) | Agricultural | |  |  |
|  | 14.6 | Fields seasonally flooded (m) | Flooded_fields | |  |  |
|  | 14.7 | Concrete dock (m) | Concrete_dock | |  |  |
|  | 14.8 | Sand outflow (m) | Sand_outflow | |  |  |
|  | 14.9 | Infractures (m) | Infra | |  |  |
|  | 14.10 | Rock, infractures at 15m (m) | Rock_15m | |  |  |
|  | 14.11 | Outflow (m) | Outflow | |  |  |
|  | 14.12 | Concrete wall (m) | Wall | |  |  |
|  | 14.13 | Concrete wall and dock (m) | Wall_dock | |  |  |
|  | 14.14 | Cobbles (m) | Cobbles | |  |  |
|  | 14.15 | Concrete structure (m) | Concrete | |  |  |
|  | 14.16 | Rocks, mine (m) | Rocky_mine | |  |  |
|  | 14.17 | Rocks, roadbank (m) | Rocky_roadbank | |  |  |
|  | 14.18 | Roadbank, settlement (m) | Roadbank_settle | |  |  |
|  | 14.19 | Structure (m) | Structure | |  |  |
|  | 14.20 | Road (m) | Road | |  |  |
|  | 14.21 | Roadbank (m) | Roadbank | |  |  |
|  | 14.22 | Dock, port (m) | Dock_Port | |  |  |
|  | 14.23 | Construct (m) | Construct | |  |  |
|  | 14.24 | National waters (m) | National_water | |  |  |
|  | 14.25 | Seasonally flooded fields and settlement (m) | Flooded_fields_settle | |  |  |
|  | 14.26 | Concrete wall (in front has a row of rocks with vegetation, m) | Wall_veg | |  |  |
|  | 14.27 | Natural areas (with structures, m) | Natural_baths | |  |  |
|  | 14.28 | Sand with infractures (m) | Sand_infra | |  |  |
|  | 14.29 | Rocks, water level fluctuation (m) | Rocky_WLF | |  |  |
|  | 14.30 | Rocks with vegetation and roadbank (m) | Rocky_Veg_Roadbank | |  |  |
|  | 14.31 | Channel outflow (m) | Channel_Out | |  |  |
|  | 14.32 | Concrete dock with rocks (m) | Dock_Rock | |  |  |
|  | 14.33 | Concrete structure (dock, m) | Concrete_pier | |  |  |
|  | 14.34 | Construction (reservoir, m) | Reservoir | |  |  |
|  | 14.35 | Sand (water level fluctuation, shrubs, m) | Sand_WLF | |  |  |
|  | **15. Catchment area (km^2^, EKBY)** | | | **Basin** | | |
|  | **16.Lake area/catchment area (%)** | | | **Per_Area_basin** | | |
|  | **17. Percentage of corine land cover in a buffer zone of 100 m (CLC, 2018)** | | | | | |
|  | 17.1 | Urban fabric (%) | Per_Urban | |  |  |
|  | 17.2 | Industrial, commercial and transport units (%) | Per_Indu | |  |  |
|  | 17.3 | Artificial, non-agricultural areas vegetated area (%) | Per_technical | |  |  |
|  | 17.4 | Mine, dump and construction sites (%) | Per_mine | |  |  |
|  | 17.5 | Arable land (%) | Per_agri | |  |  |
|  | 17.6 | Permanent crops (%) | Per_perm_agri | |  |  |
|  | 17.7 | Pastures (%) | Per_Pastures | |  |  |
|  | 17.8 | Fallow land (%) | Per_Fallow | |  |  |
|  | 17.9 | Forests (%) | Per_Fore | |  |  |
|  | 17.10 | Shrub and/or herbaceous vegetation associations (%) | Per_shrubs | |  |  |
|  | 17.11 | Open spaces with little or no vegetation (%) | Per_low_veg | |  |  |
|  | 17.12 | Inland wetlands (%) | Per_inland | |  |  |
|  | 17.13 | Maritime wetlands (%) | Per_sea_wet | |  |  |
|  | 17.14 | Marine waters (%) | Per_Sea | |  |  |
|  | **18. Structures in the littoral area (Mavromati et al., 2023)** | | | | | |
|  | 18.1 | Marina (km) | Marine | | |  |
|  | 18.2 | Beach (km) | Beach | | |  |
|  | 18.3 | Natural (km) | Natural | | |  |
|  | **19. Modified shoreline length (%, Mavromati et al., 2023)** | | **Per_mod_shore** | | |  |
|  | **20. Non-natural land cover (%) within the 100m buffer zone (Categories 1 & 2; CLC, 2018)** | | **Per_non_natural** | | |  |
|  | **21. Natural land cover (%) within the 100m buffer zone (Categories 3, 4 & 5; CLC, 2018)** | | **Per_natural** | | |  |
|  | **22. Non-artificial land cover (%) within the 100m buffer zone (Categories 2, 3, 4 & 5; CLC, 2018)** | | **Per_non_artif** | | |  |
|  | **23. Coverage of macrophytes in the littoral zone (%; EEA, 2020)** | | **Per_macro_veg** | | |  |
|  | **24. Species composition and diversity of macrophytes (EEA, 2020)** | | **Nb_Macro** | | |  |
| **Hydrological parameters** | **25. Volume (m^3^; ΕΚΒΥ, 2021)** | | **Volume** | | |  |
|  | 25.1 Volume deviation (m^3^) | | Volume_dev | | |  |
|  | 25.2 Volume deviation (%) | | Per_volume_dev | | |  |
|  | **26. Retention time (yr; ΕΚΒΥ, 2021)** | | **Retention** | | |  |
|  | **27.1 Absolute elevation of water level (**amsl)**; ΕΚΒΥ, 2021)** | | **Wlevel** | | |  |
|  | 27.2 Absolute elevation of water level (amsl) in warm period | | Wlevel_warm | | |  |
|  | 27.3 Deviation of absolute elevation of water level (amsl) in warm period | | Wlevel_warm_dev | | |  |
|  | 27.4 Percentage deviation of absolute elevation of water level (amsl) in warm period | | Per_Wlevel_warm_dev | | |  |
|  | 27.5 Absolute elevation of water level (amsl) in cold period | | Wlevel_cold | | |  |
|  | 27.6 Deviation of absolute elevation of water level (amsl) in cold period | | Wlevel_cold_dev | | |  |
|  | 27.7 Percentage deviation of absolute elevation of water level (amsl) during cold period | | Per_Wlevel_cold_dev | | |  |
|  | **28. Ecological water level** | | **ECL_dev** | | |  |
| **Typology** | **29. Type** | | **Type** | | |  |
|  | **30. Geology** | | **Geology** | | |  |
|  | **31. Water permeability** | | **Water_perm** | | |  |
|  | **32. Type of aquifer** | | **Type_aquifer** | | |  |
|  | **33. Stratification** | | **Stratification** | | |  |
|  | **34. Climate** | | **Climate** | | |  |

**Figure S1.** Spearman ranks correlations between morphological metrics in natural lakes. Abbreviations are explained in Table S1.

**Figure S2.** Spearman ranks correlations between hydrological metrics in natural lakes. Abbreviations are explained in Table S1.

**Figure S3.** Spearman rank correlations between morphological metrics in reservoirs. Abbreviations are explained in Table S1.

**Figure S4.** Spearman rank correlations between hydrological metrics in reservoirs. Abbreviations are explained in Table S1.

|  |  |  |
| --- | --- | --- |
|  |  |  |
|  |  |  |
|  |  |  |
|  |  |  |
|  |  |  |
|  |  |  |
|  |  |  |
|  |  |  |
|  |  |  |
|  |  |  |
|  |  |  |
|  |  |  |
|  |  |  |
|  |  |  |
|  |  |  |
|  |  |  |
|  |  |  |
|  |  |  |
|  |  |  |
|  |  |  |
|  |  |  |

**Figure S5.** The box plots with morphological and hydrological metrics for natural lakes. Solid lines indicate median values, boxes represent interquartile ranges (25–75%), outer lines the highest and lowest values, circle (○) the outlier values and asterisk (*) extreme values. Abbreviations are explained in Table S1.

.

|  |  |  |
| --- | --- | --- |
|  |  |  |
|  |  |  |
|  |  |  |
|  |  |  |
|  |  |  |
|  |  |  |
|  |  |  |

**Figure S6.** The box plots with morphological and hydrological metrics for artificial lakes. Solid lines indicate median values, boxes represent interquartile ranges (25–75%), outer lines the highest and lowest values, circle (○) the outlier values and asterisk (*) extreme values. Abbreviations are explained in Table S1.
